# Sequential SDF-1/CGRP-releasing smart composite hydrogel promotes osteoporotic fracture healing by targeting sensory nerve-regulated bone remodeling

**DOI:** 10.1016/j.mtbio.2025.101750

**Published:** 2025-04-17

**Authors:** Yuan Wang, Zhen Pan, Qianliang Wang, Yuexia Shu, Zhenyu Tan, Yujie Chen, Jieming He, Jia Wang, Jielin Wang, Jun Yan

**Affiliations:** a Department of Orthopedics, The Second Affiliated Hospital of Soochow University, Suzhou, 215004, China; b Department of Orthopedics, TongRen Hospital, School of Medicine Shanghai Jiao Tong University, Shanghai, 200336, China; c Department of Orthopedics, Shanghai Jiao Tong University Affiliated Sixth People's Hospital, Shanghai, 200235, China; d Department of Pathology, Tongji Hospital, Tongji University, Shanghai, 200065, China; e Laboratory of Key Technology and Materials in Minimally Invasive Spine Surgery, Tongren Hospital, Shanghai Jiao Tong University School of Medicine, Shanghai, 200336, China; f Center for Spinal Minimally Invasive Research, Shanghai Jiao Tong University, Shanghai, 200336, China

**Keywords:** Osteoporotic fractures, Smart hydrogel, Sensory nerves, Callus remodeling, Fracture healing

## Abstract

Osteoporotic fractures typically exhibit delayed healing due to impaired cell recruitment, chronic inflammation, and disrupted neurovascular signaling. Sensory nerve signaling plays a crucial role in fracture repair, and its deficiency is a significant factor leading to delayed healing. Addressing these deficiencies is crucial to overcoming the challenges associated with delayed bone repair in osteoporosis. In this study, a smart composite hydrogel (denoted as OCS-MPC) was synthesized by embedding CGRP-functionalized polydopamine-coated MXene nanosheets (MXene/PDA/CGRP) into boronic acid-modified oxidized hyaluronic acid-crosslinked carboxymethyl chitosan (OHA-PBA/CMCS) hydrogel loaded with SDF-1. OCS-MPC hydrogel enables the controlled release of SDF-1 and CGRP, aiming to promote early callus formation and late-stage callus remodeling in osteoporotic fractures. Due to dynamic crosslinking via imine and borate ester bonds, OCS-MPC exhibits rapid gelation, injectability, and self-healing properties. In vitro experiments demonstrated excellent osteogenic, angiogenic, and neurogenic properties of OCS-MPC hydrogel. In vivo studies using an osteoporotic femoral fracture model showed that OCS-MPC hydrogel enhanced MSCs recruitment via the SDF-1/CXCR4 signaling axis, significantly improving callus formation in the early stages of fracture repair. Additionally, OCS-MPC hydrogel significantly promoted callus mineralization and remodeling in the later stages of osteoporotic fracture healing through enhancing CGRP signaling. Immunofluorescence analysis further confirmed increased expression of TUBB3, CGRP, and CD31, indicating successful regeneration of the neurovascular network. These findings highlight the potential of OCS-MPC hydrogel in addressing both early and late-stage challenges of osteoporotic fracture healing, providing a promising therapeutic strategy for enhancing bone regeneration in osteoporotic patients.

## Graphical abstract

Schematic of OCS-MPC hydrogel: Burst SDF-1 release recruits MSCs early, while sustained CGRP release promotes neurovascular regulation for bone remodeling.

**Figure:**
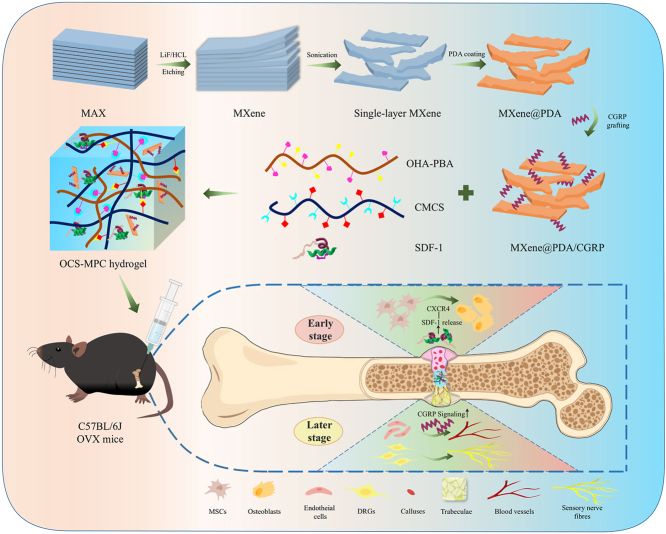

## 1 Introduction

As the population ages, the incidence of delayed fracture healing and nonunion due to osteoporosis is rising annually [1]. The absence of effective strategies to promote fracture healing has emerged as a major challenge in addressing these issues. In osteoporotic fractures, cellular senescence and disrupted intercellular communication hinder the normal fracture repair process [2,3]. The use of stem cell-based biomaterials has not yielded the expected results, highlighting the complexity of tissue repair and regeneration mechanisms [4]. In recent years, the role of sensory nerve fibers in fracture repair has garnered increasing attention. Studies have demonstrated that sensory nerve signaling is extensively involved in fracture repair, regulating osteoblasts, osteoclasts, the immune microenvironment, and angiogenesis, thereby playing a critical role [5]. Therefore, developing bone repair strategies that mimic the normal fracture healing process through sensory nerve regulation holds significant potential.

Sensory nerve signaling is integral to the fracture repair process, playing a crucial guiding role in healing [6]. Recent studies indicate that during fracture repair, nerve growth factor is significantly upregulated after stress fractures, activating TrkA signaling from sensory nerves [7]. More importantly, blocking sensory nerve signaling impairs the reestablishment of the vascular-neural network at the fracture site, delaying callus mineralization. Our previous research identified the absence of CGRP (calcitonin gene-related peptide) signaling as a key factor hindering callus remodeling in osteoporotic fracture repair [8]. This suggests that leveraging sensory nerve signaling during the later stages of fracture repair may promote callus remodeling. Additionally, CGRP, a bioactive peptide, has been widely shown to promote angiogenesis and regulate the immune microenvironment [9]. However, the short half-life of CGRP and its rapid metabolism limit the use of exogenous CGRP [10]. Therefore, developing a suitable carrier to extend CGRP activity and precisely control its release is of great clinical significance for exploring CGRP-based sensory nerve signaling to accelerate osteoporotic fracture healing.

During fracture repair, the extracellular matrix (ECM) plays a crucial role in facilitating the exchange of signals and substances between newly formed and existing tissues [11]. The ECM provides structural support for resident stem cells and creates a complex biochemical and biomechanical signaling environment, collectively regulating stem cell self-renewal, proliferation, and differentiation [12]. In fracture healing following the endochondral ossification pathway, early aggregation of various cells at the fracture site is a critical step in initiating callus formation. Mesenchymal stem cells (MSCs) aggregate within the ECM to form cell condensations, gradually differentiating into chondrocytes through intercellular interactions, thereby forming the core of endochondral ossification [13]. Additionally, the ECM contains abundant proteoglycans and fibronectin, providing a physical scaffold for cell growth and acting as a reservoir for growth factors that regulate cell adhesion, growth, differentiation, and migration. Under osteoporotic conditions, the ECM undergoes significant changes, greatly impacting on the proliferation, differentiation, and communication of cells involved in fracture repair. Hydrogels form three-dimensional network structures through various cross-linking reactions, maintaining a hydrated state suitable for cell growth and bioactive molecule transfer [14]. Due to their excellent biocompatibility and ability to incorporate bioactive signals, hydrogels are considered capable of replicating the ECM environment needed for tissue repair [15]. Therefore, hydrogel-based bone repair strategies hold significant potential for clinical applications.

In this study, we developed a smart composite hydrogel named OCS-MPC by embedding CGRP-functionalized polydopamine-coated MXene nanosheets (MXene/PDA/CGRP, MPC) into a boronic acid-modified oxidized hyaluronic acid-crosslinked carboxymethyl chitosan (OHA-PBA/CMCS, OCS) hydrogel loaded with SDF-1 (stromal cell-derived factor 1). In the OCS-MPC hydrogel system, OHA-PBA and CMCS formed a dual chemical network with dynamic cross-linking through imine bonds and boronate ester bonds, providing rapid gelation, injectability, and excellent self-healing properties.

The weak interactions between SDF-1 and the hydrogel facilitated the rapid release of SDF-1 in the early stages of fracture repair, enhancing the homing ability of mesenchymal stem cells (MSCs) and accelerating callus formation. As the hydrogel degraded, CGRP-loaded nanomaterials were gradually exposed to the fracture repair microenvironment. During the mid-to-late repair stages, CGRP and sensory neural signaling regulated callus remodeling, mimicking the natural fracture healing process to promote osteoporotic fracture repair. This composite hydrogel was designed for the sequential release of SDF-1 and CGRP, achieving spatiotemporal regulation of osteoporotic fracture healing. This study comprehensively investigated the effects and mechanisms of OCS-MPC in osteoporotic fracture healing at material, molecular, cellular, and animal levels, providing a novel strategy for osteoporotic fracture treatment.

## 2 Results

### 2.1 Preparation and characterization of MXene@PDA and OCS-MPC hydrogel

To address the challenges of impaired early callus formation and delayed mineralization in osteoporotic fractures, a novel OHA-PBA/CMCS/SDF-1/MXene@PDA/CGRP(OCS-MPC) dual-crosslinked hydrogel was designed and developed. This hydrogel aims to achieve multiple regulatory functions, including burst release of SDF-1 to promote cell recruitment and sustained release of CGRP to support neurovascular reconstruction and bone repair (Graphical abstract). This design offers an efficient solution for osteoporotic fracture healing.

**Fig. 1:**
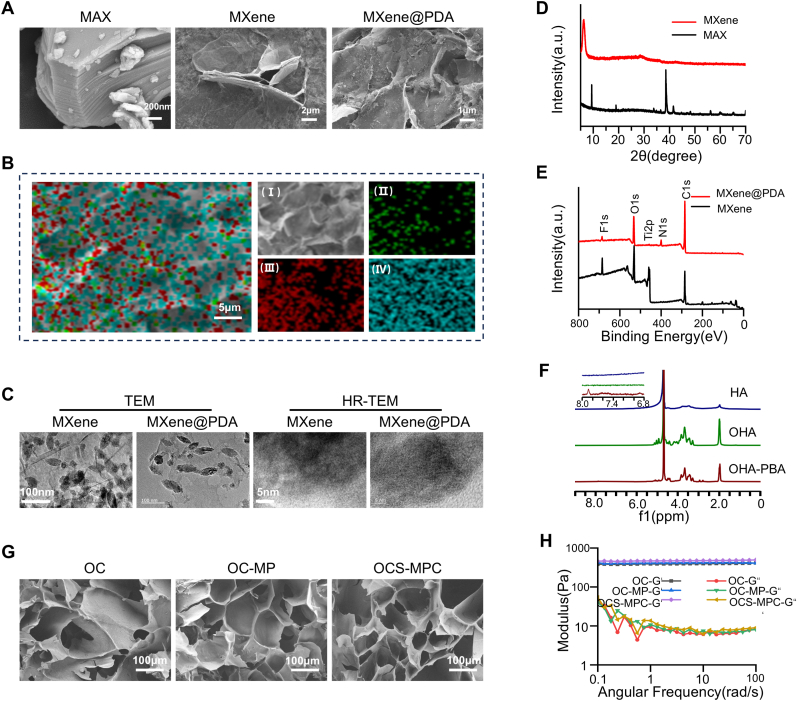
Characterization of MXene@PDA and OCS-MPC (A) SEM images of MAX, monolayer MXene, and MXene@PDA. (B) EDS elemental mapping of MXene@PDA (scale bar = 5 μm). (C) TEM and HR-TEM images of MXene and MXene@PDA (left scale bar = 100 nm; right scale bar = 5 nm). (D) XRD spectra of MAX and MXene. (E) XPS spectra of MXene and MXene@PDA. (F) ^1^H NMR spectra of HA, OHA, and OHA-PBA. (G) SEM images of OC, OC-MP, and OCS-MPC (scale bar = 100 μm). (H) Storage modulus (G′) and loss modulus (G″) of OC, OC-MP, and OCS-MPC as a function of angular frequency. All data are representative of at least three independent experiments. Data are presented as mean ± SEM. ∗P < 0.05, ∗∗P < 0.01, ∗∗∗P < 0.001, ∗∗∗∗P < 0.0001.

CGRP's application is limited by its low stability and short half-life in vivo [16]. To improve CGRP stability and extend its release duration, polydopamine (PDA) was first functionalized and coated onto MXene nanosheets to construct MXene@PDA/CGRP(MPC). MAX (Ti3AlC2) was etched with HCl/LiF to remove the aluminum layer, producing multilayer MXene (Ti3C2) [17]. Scanning electron microscopy (SEM) images showed a typical layered structure resembling an accordion (Fig. 1A). Since multilayer MXene is too large for nanomedical applications, single-layer MXene was prepared by ultrasonication, resulting in a two-dimensional layered structure with a size of 2–5 μm and a smooth surface (Fig. 1A). To enhance the adsorption capacity of MXene for CGRP, PDA was coated onto its surface to form MXene@PDA. SEM images showed evidence of polymer coating on the MXene@PDA surface, with no significant change in overall size (Fig. 1A). Energy-dispersive spectroscopy (EDS) analysis confirmed the distribution of Ti and N elements on the MXene@PDA surface, verifying the formation of a uniform PDA coating on MXene (Fig. 1B). Transmission electron microscopy (TEM) was used to further characterize the structural properties of MXene and MXene@PDA (Fig. 1C). The results indicated that MXene had a sheet-like structure approximately 100–200 nm in size. In contrast, MXene@PDA retained its layered structure but appeared darker, indicating increased thickness, with a distinct outer boundary due to successful PDA coating. These results demonstrate the successful construction of CGRP-functionalized MXene@PDA nanosheets with favorable physicochemical properties. X-ray diffraction (XRD) was used to further analyze the crystal structure of MAX, MXene, and MXene@PDA. The results showed a strong (104) peak at 2θ ≈ 39° in MAX, which disappeared in MXene, whereas the (002) peak shifted from 2θ ≈ 9.4° to 2θ ≈ 6.2° (Fig. 1D). These changes confirm the successful removal of the aluminum layer and preparation of MXene. X-ray photoelectron spectroscopy (XPS) was used to analyze the chemical composition of MXene and MXene@PDA. The results indicated that the MXene@PDA surface had newly introduced N elements, while the Ti and F contents decreased, and C and O contents increased (Fig. 1E). These results were confirmed by fine peak fitting of the MXene@PDA spectra, where the appearance of the N 1s peak directly indicated successful modification of MXene with polydopamine (Fig. S1A).

To enhance cell recruitment and signal exchange during the early stages of fracture repair, an OHA-PBA/CMCS hydrogel containing SDF-1 was synthesized (Fig. S1B and C). Successful grafting of PBA onto OHA was confirmed by ^1^HNMR spectra, which showed new characteristic peaks at 2.80, 2.96, and 7.88 ppm (Fig. 1F). Subsequently, MXene@PDA/CGRP and SDF-1 were mixed into a 5 % carboxymethyl chitosan (CMCS) and OHA-PBA solution to successfully prepare the OCS-MPC hydrogel. To evaluate the effect of MXene@PDA incorporation on hydrogel gelation and mechanical properties, OHA-PBA/CMCS hydrogel (OC) and OHA-PBA/CMCS/MXene@PDA hydrogel (OC-MP) was prepared as controls. SEM analysis showed that the pore size of OCS-MPC hydrogel was approximately 154 μm, with no significant change compared to controls OC (158 μm) and OC-MP (161 μm), indicating that adding MXene@PDA/CGRP had minimal effect on the hydrogel's microporous structure (Fig. 1G). SEM revealed that OCS-MPC hydrogel had a uniform microporous structure, providing a stable microenvironment for cell growth (Fig. 1G). Rheological testing showed that OCS-MPC hydrogel exhibited similar elasticity and viscoelasticity compared to the control hydrogels, demonstrating excellent mechanical stability for biomedical applications (Fig. 1H). These results indicate that the OCS-MPC hydrogel has excellent physicochemical properties, making it a promising material for osteoporotic fracture repair.

### 2.2 Properties of OCS-MPC hydrogel

**Fig. 2:**
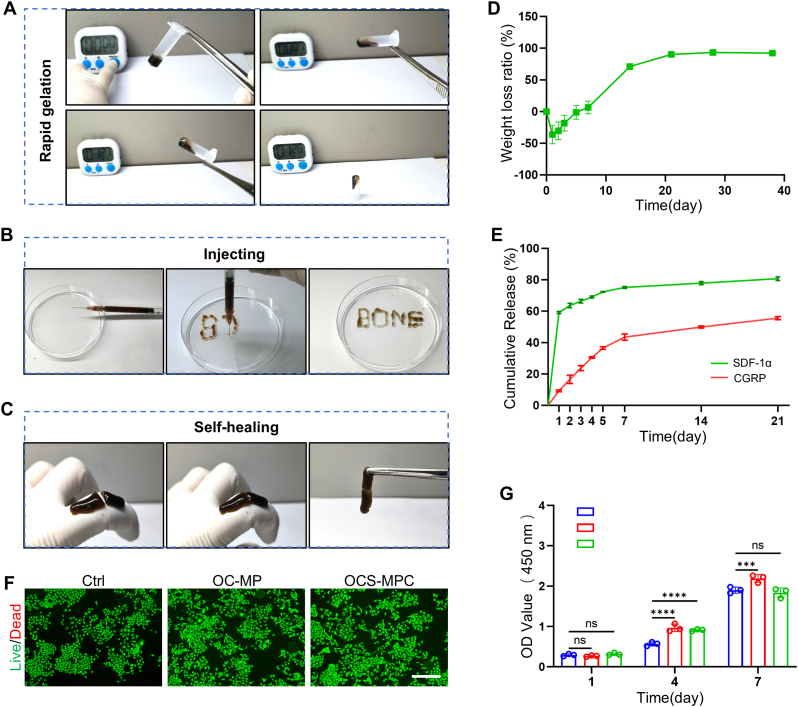
Properties of OCS-MPC Hydrogel (A) Rapid gelation performance of OCS-MPC. (B) Injectable properties of OCS-MPC. (C) Self-healing properties of OCS-MPC. (D) Degradation rate of OCS-MPC under simulated physiological conditions. (E) Cumulative release of SDF-1 and CGRP from OCS-MPC (n = 3). (F) Representative calcein-AM/PI live/dead staining images (scale bar = 200 μm). (G) CCK-8 assay results on days 1, 4, and 7 (n = 3). All data are representative of at least three independent experiments. Data are presented as mean ± SEM. ∗P < 0.05, ∗∗P < 0.01, ∗∗∗P < 0.001, ∗∗∗∗P < 0.0001.

Osteoporotic fractures are often accompanied by comminution and bone defects, complicating anatomical reduction during surgical treatment. Excessive fracture gaps severely impact fracture healing [18]. Therefore, the newly developed OCS-MPC hydrogel should possess injectability, rapid gelation, and self-healing properties to quickly fill fracture gaps and facilitate bone healing. To this end, these properties of the OCS-MPC hydrogel were evaluated. When equal volumes of 5 % CMCS and 5 % OHA-PBA were mixed, the mixture lost fluidity within approximately 30 s and rapidly formed a hydrogel (Fig. 2A). This rapid gelation allows for immediate filling of the fracture site. To verify the injectability of OCS-MPC hydrogel, an injection experiment was conducted using a 1 ml syringe (Fig. 2B). The results showed that OCS-MPC hydrogel solidified and shaped quickly after injection, forming the desired morphology. Additionally, the self-healing properties of OCS-MPC hydrogel were evaluated. After cutting the hydrogel, the cut surfaces were brought into close contact, and the hydrogel reconnected quickly, maintaining its integrity (Fig. 2C). These results indicate that OCS-MPC hydrogel possesses excellent rapid gelation, injectability, and self-healing properties, making it well-suited for clinical treatment of osteoporotic fractures.

Next, the degradation characteristics, release behavior of factors, and biocompatibility of the OCS-MPC hydrogel were evaluated. The swelling ratio is a key parameter reflecting the hydrogel's ability to exchange nutrients and gases. The results indicated that OCS-MPC hydrogel reached swelling equilibrium after 24 h, with a swelling ratio of 178.1 ± 5.2 %, suggesting good nutrient and gas exchange capabilities (Fig. 2D). The OCS-MPC hydrogel increased in weight during the initial swelling stage, then gradually degraded and was fully degraded within approximately one month, aligning with the natural fracture healing process (Fig. 2D). To evaluate the release behavior of SDF-1 and CGRP, their cumulative release was measured using ELISA. The results indicated that SDF-1 exhibited burst release in the first 7 days, while CGRP was released slowly and continuously over a month (Fig. 2E). This sequential release pattern aligns with the physiological needs of different stages of fracture repair. For biocompatibility evaluation, the toxicity of OCS-MPC hydrogel to HUVECs was assessed using Calcein-AM/PI (live/dead) staining and CCK8 assays. The live/dead staining results indicated that OCS-MPC hydrogel had good biocompatibility with HUVECs, with almost no dead cells observed under fluorescence microscopy (Fig. 2F–S1D-E). The CCK8 assay results further supported this finding, suggesting that the hydrogel had no significant toxicity to cell proliferation (Fig. 2G). In summary, OCS-MPC hydrogel exhibits excellent properties, including rapid gelation, injectability, self-healing, and good biocompatibility. It also allows for programmable release of SDF-1 and CGRP, providing an effective material solution for osteoporotic fracture repair. Its multifunctional characteristics make it a promising material for bone repair.

### 2.3 Evaluation of In Vitro Osteogenic Properties of OCS-MPC hydrogel

**Fig. 3:**
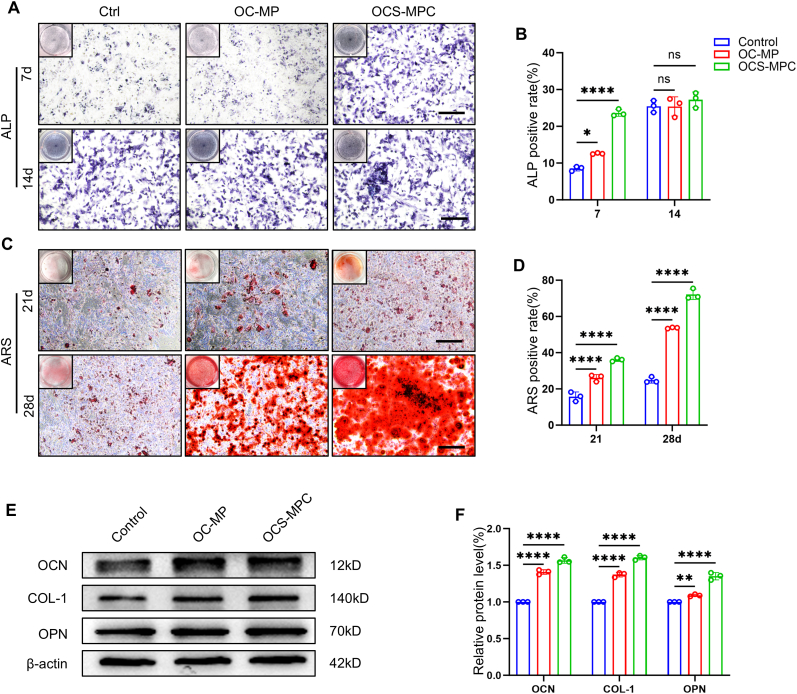
In Vitro Osteogenic Properties of OCS-MPC Hydrogel (A) Representative ALP staining images of BMSCs treated with OC-MP and OCS-MPC after 7 and 14 days. (B) Quantitative analysis of (A). (C) Representative ARS staining images of MSCs treated with OC-MP and OCS-MPC after 21 and 28 days. (D) Quantitative analysis of (C). (E) Western blot analysis of osteogenic proteins (OCN, COL-1, and OPN) in BMSCs cultured with OC-MP and OCS-MPC. (F) Quantitative analysis of osteogenic protein expression using ImageJ. Scale bar = 200 μm. n = 3 per group. All data are representative of at least three independent experiments. Data are presented as mean ± SEM. ∗P < 0.05, ∗∗P < 0.01, ∗∗∗P < 0.001, ∗∗∗P < 0.0001.

In bone repair via endochondral ossification, osteogenic differentiation of bone marrow mesenchymal stem cells (BMSCs) is a key indicator of the osteogenic potential of a material. Therefore, alkaline phosphatase (ALP) and Alizarin Red S(ARS) staining were used to evaluate the effect of OCS-MPC hydrogel on osteogenic differentiation. On day 7 of osteogenic induction, ALP staining indicated that ALP activity in the OC-MP and OCS-MPC groups was significantly higher than that in the control group (Fig. 3A). Quantitative analysis indicated that ALP activity in the OC-MP and OCS-MPC groups was 1.5 times and 2.8 times that of the control group, respectively (Fig. 3B). The ALP activity in the OCS-MPC group was significantly higher than in the other two groups, suggesting that the burst release of SDF-1 from the OCS-MPC hydrogel effectively promoted osteogenic differentiation in the early stage. By day 14 of osteogenic induction, there was no significant difference in ALP activity among the three groups (Fig. 3A and B), indicating that the promoting effects of each group were similar in the middle stage of osteogenic differentiation.

To further evaluate mineralization, ARS staining was performed on day 21 of osteogenic induction. The results indicated that both OC-MP and OCS-MPC groups exhibited higher mineralized nodule formation compared to the control group, with significantly enhanced ARS-positive areas in the OCS-MPC group (Fig. 3C). Quantitative analysis indicated that the ARS-positive rate in the OCS-MPC group was 2.3 times and 1.4 times that of the control and OC-MP groups, respectively (Fig. 3D). At day 28, ARS staining results continued to indicate a significant mineralization-promoting effect in the OCS-MPC group, consistent with the trend observed on day 21 (Fig. 3C and D). These results suggest that the sustained release of CGRP from the OCS-MPC hydrogel significantly promoted late-stage osteogenic mineralization.

Additionally, qPCR and Western Blot (WB) were used to measure the expression of osteogenic differentiation-related genes and proteins. The results indicated that the expression levels of OCN (osteocalcin), COL-1 (type I collagen), and OPN (osteopontin) genes and proteins were significantly higher in the OCS-MPC group compared to the other two groups (Fig. 3E–F, S2A). The high expression of these molecular markers further confirmed the promoting effect of OCS-MPC hydrogel on osteogenic differentiation, consistent with ALP and ARS staining results. In summary, the OCS-MPC hydrogel demonstrated significant promoting effects at various stages of osteogenic differentiation through early burst release of SDF-1 and sustained release of CGRP. Its excellent in vitro osteogenic properties lay a solid foundation for further in vivo research, highlighting its potential as a bone repair material.

### 2.4 Evaluation of In vitro angiogenesis and nerve regeneration potential of the OCS-MPC hydrogel

**Fig. 4:**
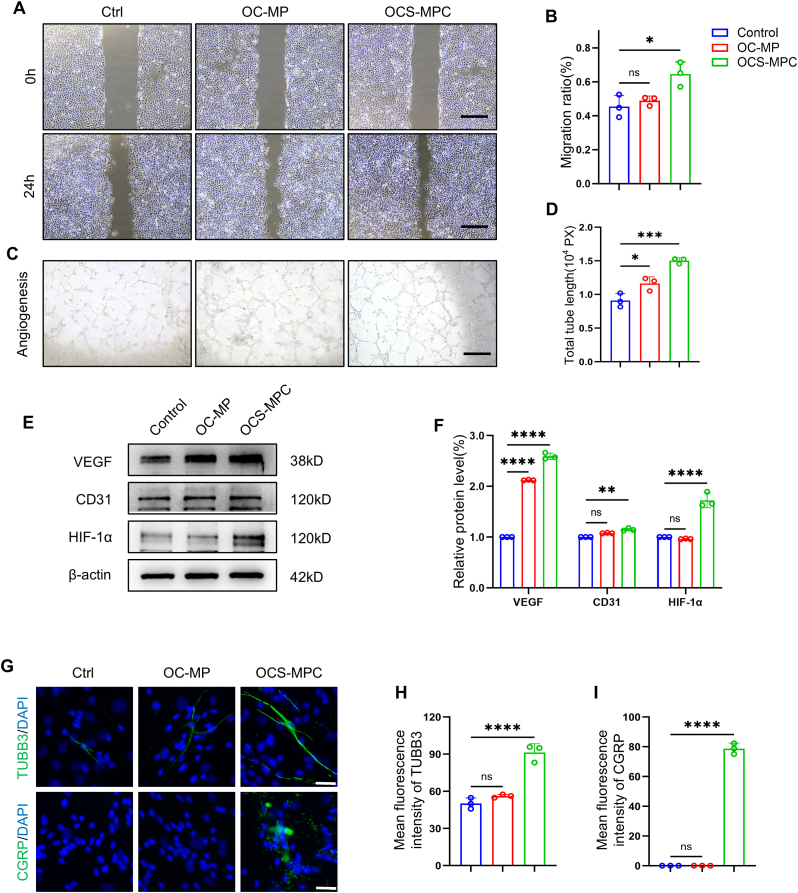
Pro-Angiogenic and Neurogenic Properties of OCS-MPC Hydrogel In Vitro (A) Representative scratch assay images of HUVECs treated with OC-MP and OCS-MPC for 24 h (scale bar = 200 μm). (B) Quantitative analysis of wound closure area using ImageJ (n = 3). (C) Representative tube formation assay images of HUVECs treated with OC-MP and OCS-MPC (scale bar = 200 μm). (D) Quantitative analysis of total tube length and number of branches using ImageJ (n = 3). (E) Western blot analysis of angiogenic proteins (VEGF, CD31, and HIF-1α) in HUVECs cultured with OC-MP and OCS-MPC. (F) Quantitative analysis of angiogenic protein expression using ImageJ (n = 3). (G) Representative immunofluorescence images of TUBB3 and CGRP in DRGs treated with OC-MP and OCS-MPC (scale bar = 50 μm). (H–I) Quantitative analysis of TUBB3 and CGRP protein expression levels (n = 3). All data are representative of at least three independent experiments. Data are presented as mean ± SEM. ∗P < 0.05, ∗∗P < 0.01, ∗∗∗P < 0.001, ∗∗∗∗P < 0.0001.

Vascular and nerve regeneration are crucial for successful fracture healing. Therefore, HUVECs were used to conduct scratch wound assays, Transwell migration assays, and tube formation assays to evaluate the in vitro pro-angiogenic properties of the OCS-MPC hydrogel. In the scratch wound assay, all experimental groups showed the same scratch width at 0 h. After 24 h, the scratch width in the OCS-MPC group was significantly reduced compared to that in the control and OC-MP groups (Fig. 4A). Quantitative analysis indicated that the migration rate of HUVECs in the OCS-MPC group was 1.4 times that of the control group and 1.3 times that of the OC-MP group, suggesting that OCS-MPC hydrogel significantly accelerated HUVEC migration (Fig. 4B). Similarly, the Transwell migration assay confirmed that OCS-MPC hydrogel accelerated cell migration, with the number of migrated cells in the OCS-MPC group being 1.6 times that in the control group and 1.2 times that in the OC-MP group (Fig. S2B–C). To further evaluate the effect of OCS-MPC hydrogel on angiogenesis, an in vitro tube formation assay was performed. As shown in Fig. 4C, more pronounced tubular structures were observed in the OCS-MPC group. Quantitative analysis indicated that the number of branch points and the total tube length formed in the OCS-MPC group were significantly higher than those in the control and OC-MP groups (Fig. 4D–S2D). Additionally, the expression of angiogenesis-related genes and proteins, including VEGF, CD31, and HIF-1α, was significantly increased in the OCS-MPC group (Fig. 4E–F, S2E), further confirming its pro-angiogenic ability.

Next, the effect of OCS-MPC hydrogel on nerve regeneration was evaluated. OCS-MPC hydrogel was co-cultured with dorsal root ganglia (DRGs) from mice, and its effect on nerve differentiation and neurite growth was assessed using immunofluorescence staining. The results indicated that after 10 days of nerve differentiation induction, the axon length of DRGs in the OCS-MPC group significantly increased, and the expression of TUBB3 (neuron-specific protein β-III tubulin) was significantly enhanced (Fig. 4G and H). These findings suggest that the OCS-MPC hydrogel effectively promotes neurite outgrowth and nerve differentiation. Since calcitonin gene-related peptide (CGRP), secreted by sensory nerves, plays an important role in bone healing, immunofluorescence staining for CGRP was also performed. The results indicated that no significant CGRP expression was observed in the control or OC-MP groups, whereas CGRP protein expression was significantly enhanced in the OCS-MPC group (Fig. 4G and I). These findings suggest that the OCS-MPC hydrogel effectively promotes the secretion of the neurotransmitter CGRP, which may regulate the bone repair process via neural signaling. In summary, the OCS-MPC hydrogel demonstrated significant in vitro angiogenic and neurogenic properties by promoting angiogenesis, accelerating nerve growth, and enhancing CGRP secretion. These results provide a solid foundation for osteoporotic fracture repair, facilitating effective intercellular signaling and thereby accelerating bone healing.

### 2.5 OCS-MPC hydrogel accelerates callus formation in early stage of fracture repair via the SDF-1/CXCR4 axis

**Fig. 5:**
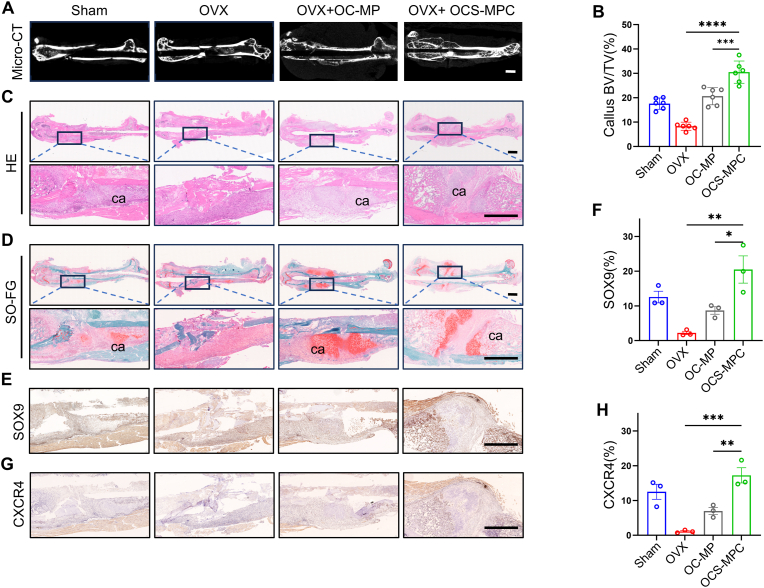
OCS-MPC Promotes Callus Formation in Early-Stage Osteoporotic Fracture Healing via the SDF-1/CXCR4 Axis (A–B) Representative micro-CT images and quantification of femoral fractures in mice treated with OC-MP or OCS-MPC at 1-week post-fracture. Parameters include bone volume fraction (BV/TV). (Scale bar = 1 mm. n = 6). (C–D) Representative HE (C) and SO-FG (D) stained images of femoral fractures in mice treated with OC-MP or OCS-MPC at 1-week post-fracture. Upper panels show global views; lower panels show close-ups of the fracture sites. ca: callus. (Scale bar = 1 mm). (E–F) Immunohistochemical staining images and quantification of SOX9 protein levels in calluses from mice treated with OC-MP or OCS-MPC at 1-week post-fracture. (Scale bar = 1 mm. n = 3). (G–H) Immunohistochemical staining images and quantification of CXCR4 protein levels in calluses from mice treated with OC-MP or OCS-MPC at 1-week post-fracture. (Scale bar = 1 mm. n = 3). All data are representative of at least three independent experiments. Data are presented as mean ± SEM. ∗P < 0.05, ∗∗P < 0.01, ∗∗∗P < 0.001, ∗∗∗∗P < 0.0001.

Based on the in vitro study results, the repair effects of OCS-MPC hydrogel in femoral fractures of OVX model mice were further evaluated. Early callus formation is crucial for initiating bone healing, and the timely formation of effective callus is a key factor in successful fracture repair. An OVX osteoporosis model was established by removing the bilateral ovaries of 10-week-old C57 female mice, and a femoral fracture model was constructed 8 weeks later. Micro-CT scans indicated that the amount of new callus formed in OVX mice during the first week post-fracture was significantly lower than that in the sham group. However, with the local application of OCS-MPC hydrogel at the fracture site, the amount of callus formed was similar to that in the sham group, and extensive new callus formation was observed in the OCS-MPC group (Fig. 5A, S3A). Quantitative analysis of Micro-CT data indicated that the bone volume/total volume (BV/TV) of callus in the OCS-MPC group was 5 times that in the OVX group and 2 times that in the OC-MP group (Fig. 5B), suggesting that OCS-MPC hydrogel had a significant effect on promoting early fracture repair. Histological staining further confirmed the Micro-CT results. HE and Saffron O/fast green (SO-FG) staining indicated that the OCS-MPC group formed more pronounced new cartilage callus at the fracture site compared to the other groups (Fig. 5C and D). Masson staining indicated increased collagen production at the fracture site in the OCS-MPC group (Fig. S3D), suggesting enhanced cartilage formation and tissue remodeling capacity in this group. Although the OC-MP group also promoted callus formation, its effect was inferior to that of the OCS-MPC group, consistent with the in vitro results. These findings indicate that early intercellular signaling is crucial for fracture repair, and the OCS-MPC hydrogel effectively facilitates this process.

To further evaluate the mechanism by which OCS-MPC hydrogel promotes endochondral ossification in early fracture repair, immunohistochemical staining for Sox9 and Col-2 expression was performed. The results indicated that Sox9 and Col-2 expression levels at the fracture site were significantly higher in the OCS-MPC group compared to the OVX and OC-MP groups (Fig. 5E–F, S3C), suggesting that OCS-MPC hydrogel significantly accelerated endochondral ossification by promoting chondrocyte proliferation and differentiation. Combined with the in vitro results, it was found that OCS-MPC hydrogel released SDF-1 during the early degradation stage. To investigate the mechanism of SDF-1 in promoting fracture repair, CXCR4 expression at the fracture site was further examined, and the results indicated that CXCR4 expression in the OCS-MPC group was significantly higher than in the OVX and OC-MP groups (Fig. 5G and H). These results suggest that OCS-MPC hydrogel significantly promoted cartilage callus formation through the SDF-1/CXCR4 signaling axis in early fracture repair, thereby accelerating fracture healing.

### 2.6 OCS-MPC hydrogel accelerates callus remodeling in late stage of fracture repair by promoting vascular and nerve network reconstruction

**Fig. 6:**
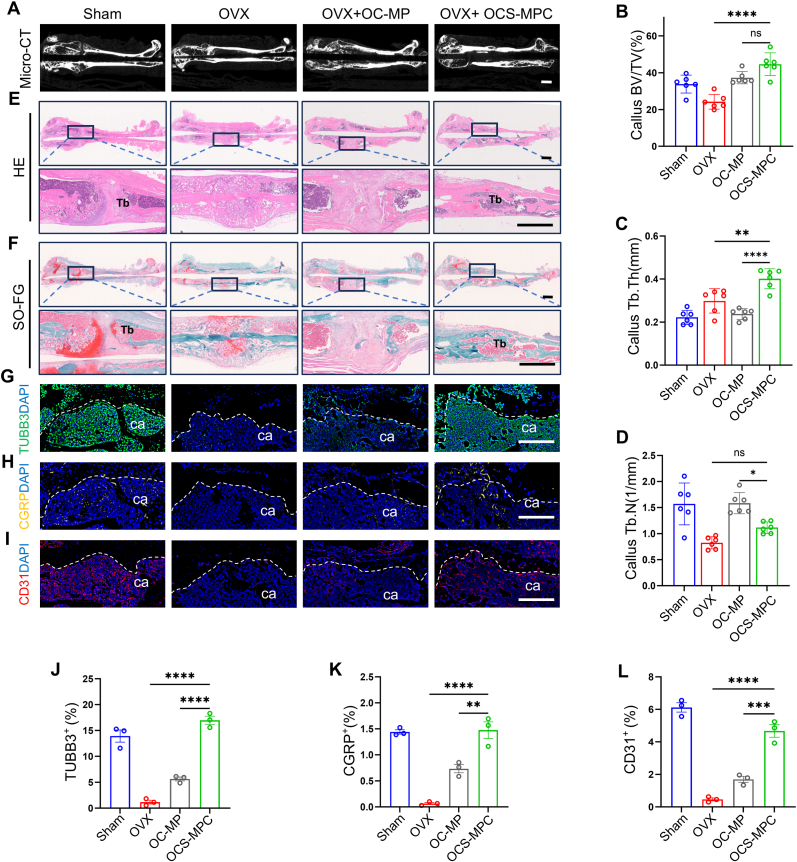
OCS-MPC Promotes Callus Remodeling in Late-Stage Osteoporotic Fracture Healing via Angioneurotic Network Reconstruction (A–D) Representative micro-CT images and quantification of femoral fractures in mice treated with OC-MP or OCS-MPC at 4 weeks post-fracture. Parameters include bone volume fraction (BV/TV), trabecular thickness (Tb.Th), and trabecular number (Tb.N). Quantification was performed using ImageJ with the CTAn plugin (n = 6). Scale bar = 1 mm. (E–F) Representative HE (E) and SO-FG (F) stained images of femoral fractures in mice treated with OC-MP or OCS-MPC at 4 weeks post-fracture. Upper panels show global views; lower panels show close-ups of the fracture sites. Tb: trabecular bone. Scale bar = 1 mm. (G and J) Immunofluorescence staining and quantification of TUBB3 protein levels in calluses from mice treated with OC-MP or OCS-MPC at 4 weeks post-fracture (n = 3). ca: callus. White dashed lines indicate callus boundaries. scale bar = 200 μm. (H and K) Immunofluorescence staining and quantification of CGRP protein levels in calluses from mice treated with OC-MP or OCS-MPC at 4 weeks post-fracture (n = 3). ca: callus. White dashed lines indicate callus boundaries. scale bar = 200 μm. (I and L) Immunofluorescence staining and quantification of CD31 protein levels in calluses from mice treated with OC-MP or OCS-MPC at 4 weeks post-fracture (n = 3). ca: callus. White dashed lines indicate callus boundaries. scale bar = 200 μm. All data are representative of at least three independent experiments. Data are presented as mean ± SEM. ∗P < 0.05, ∗∗P < 0.01, ∗∗∗P < 0.001, ∗∗∗∗P < 0.0001.

Vascularization and sensory nerve regeneration are essential for bone repair. In osteoporosis, delayed fracture healing and loss of vascular and sensory nerve signals are major reasons for poor repair. Based on this, CGRP was loaded onto MXene@PDA nanosheets within the OCS-MPC hydrogel, with the hypothesis that this material would enhance sensory nerve signaling in late fracture repair, promote crosstalk between nerves, blood vessels, and bone, and thereby accelerate callus remodeling at the fracture site. Four weeks after the fracture, micro-CT was used to evaluate the callus in osteoporotic mice. The results indicated that the rate of callus mineralization and trabecular formation in the OCS-MPC group was significantly higher than in the other groups (Fig. 6A, S3D). Quantitative analysis of micro-CT data indicated that the bone volume/total volume (BV/TV) of callus in the OCS-MPC group was 2.5 times that in the OVX group. Although there was no significant difference in trabecular number (Tb.N), trabecular thickness (Tb.Th) increased by approximately 50 %, suggesting more newly formed trabeculae and denser trabeculae per unit volume in the OCS-MPC group (Fig. 6B–D). Compared to the OC-MP group, although the size of the callus was not significantly different, the trabecular strength within the callus of the OCS-MPC group was significantly increased, indicating enhanced mineralization (Fig. 6B–D). Consistent with the micro-CT results, HE and SO-FG staining also indicated that the callus at the fracture site in the OCS-MPC group had essentially completed mineralization and remodeling (Fig. 6E and F). Masson staining further confirmed that there were more trabecular-like structures in the callus of the OCS-MPC group, suggesting more complete structural remodeling of bone tissue (Fig. S3E).

To further observe the reconstruction of the nerve-vascular network within the callus in late fracture repair, the expression of nerve and vascular marker proteins (TUBB3, CGRP, and CD31) was detected using immunofluorescence staining. TUBB3 and CGRP expression approached sham levels, significantly surpassing OVX and OC-MP groups (Fig. 6G and J, H and K. Similarly, CD31 expression, a vascular marker, was markedly elevated and close to normal levels (Fig. 6I and L). These results suggest that OCS-MPC hydrogel enhances neuro-vascular regeneration and promotes coordinated nerve-vessel-bone interactions during fracture healing. This leads to improved callus remodeling and provides a promising strategy for osteoporotic fracture treatment.

## 3 Discussion

Osteoporotic fractures with delayed healing pose a significant challenge to the health and safety of older adults [19]. Cellular senescence and chronic inflammation disrupt the bone repair process [20]. Therefore, promoting osteoporotic fracture repair as a precisely regulated process is a key goal in enhancing fracture healing. In this study, a novel injectable, self-healing OCS-MPC smart composite hydrogel was developed. CGRP-functionalized polydopamine-coated MXene nanosheets (MXene/PDA/CGRP, MPC) into a boronic acid-modified oxidized hyaluronic acid-crosslinked carboxymethyl chitosan (OHA-PBA/CMCS, OCS) hydrogel loaded with SDF-1, achieving the programmed release of SDF-1 and CGRP to mimic the natural bone healing process and promote osteoporotic fracture healing. The results demonstrated that OCS-MPC hydrogel enabled precise cytokine release in vitro, significantly promoting angiogenesis, osteogenic differentiation, DRGs nerve fiber growth. More importantly, OCS-MPC hydrogel promoted early callus formation in osteoporotic fracture repair and accelerated callus mineralization and remodeling in the later stages. Additionally, OCS-MPC hydrogel facilitated endochondral ossification by activating the SDF-1/CXCR4 signaling pathway and accelerated callus remodeling through enhanced vascular and neural network reconstruction.

Hydrogels, as natural polymers, have broad potential applications in osteoporotic fracture repair. Hyaluronic acid (HA) is a naturally occurring polysaccharide and a major component of the extracellular matrix (ECM), widely recognized as a carrier for inducing bone regeneration [21]. However, due to its high-water absorption and susceptibility to enzymatic degradation, HA hydrogel degrades too quickly in vivo, which is disadvantageous for delayed healing in osteoporotic fractures. Recent studies have shown that carboxymethyl chitosan (CMCS), a hydrophilic derivative of chitosan (CS), has received significant attention in bone tissue engineering due to its excellent properties, including high biocompatibility, biodegradability, antibacterial activity, and osteoconductivity [22,23]. Recent research found that oxidized hyaluronic acid (OHA) can be used as a crosslinker for CMCS, forming a hydrogel through Schiff base reactions between aldehyde groups in OHA and amino groups in CMCS without toxic chemical crosslinkers or irradiation sources. This hydrogel, with excellent injectability and self-healing properties, overcomes the rapid degradation disadvantage of pure HA hydrogels and has received widespread attention in fields such as postoperative adhesion prevention [24], vitreous substitutes [25], wound healing [26], and drug sustained release [27]. In this study, an OHA-PBA/CMCS hydrogel was synthesized, tailored to the acidic, hypoxic microenvironment of early osteoporotic fractures, enabling rapid gelation in the fracture gap and forming an optimal microenvironment for cell growth at the fracture site. Additionally, to overcome the limitations of weak mechanical properties and uncontrolled cytokine release in hydrogels, studies have shown that bioactive nanomaterials, such as MXenes, can be combined with hydrogels to enhance bone growth effects [28]. Zehui Lv et al. introduced BMP-2-functionalized magnesium-iron layered double hydroxide (LDH) nanosheets into a chitosan/silk fibroin (CS) hydrogel loaded with platelet-derived growth factor-BB (PDGF-BB), constructing an smart injectable temperature-responsive hydrogel (CSP-LB) that achieved burst release of PDGF-BB and sustained release of BMP-2, thereby promoting efficient bone regeneration [29]. In this study, CGRP was adsorbed onto MXene@PDA nanosheets, significantly extending the release time and activity of CGRP, thereby promoting callus mineralization and remodeling in the later stages of osteoporotic fracture healing.

During endochondral ossification, stem cells play an important role in early recruitment to the fracture site and in the formation of new cartilage calluses [30]. However, in osteoporotic fractures, stem cells exhibit significantly reduced recruitment ability due to senescence [31,32]. Previous studies have reported that removing senescent cells and metabolic byproducts can enhance the osteogenic differentiation capacity of mesenchymal stem cells [33]. Research has shown that SDF-1 can recruit stem cells to participate in tissue injury repair, and Ling et al. demonstrated the regulatory effect of the SDF-1/CXCR4 signaling axis on BMSCs migration during liver injury repair [34]. In our study, a composite hydrogel was designed to release SDF-1 into the fracture microenvironment within one week by degrading during the early phase of fracture repair. In vivo experimental results indicated that this hydrogel effectively recruited numerous mesenchymal stem cells to the fracture site in the early phase of fracture repair, providing a sufficient cell source for callus formation and significantly promoting early cartilage callus formation. CGRP plays dual roles in regulating bone remodeling by promoting osteoblast proliferation and differentiation, while simultaneously inhibiting osteoclast formation and bone resorption [9]. Previous studies have demonstrated that CGRP enhances osteogenic differentiation of BMSCs by upregulating Runx2 and BMP2 signaling pathways [35]. Meanwhile, CGRP suppresses RANKL-induced osteoclastogenesis, suggesting its potential in mitigating osteoporotic bone loss [36]. These findings are consistent with our results showing enhanced callus formation and remodeling in the CGRP-containing hydrogel group. These results validate the potential application of the OCS-MPC hydrogel in osteoporotic fracture repair, particularly in promoting early cell recruitment and new cartilage callus formation.

In the late stage of osteoporotic fracture repair, delayed mineralization of cartilage callus is a key reason for delayed fracture healing and recurrent fractures [37]. Recent studies have shown that sensory nerve fibers play an important role in fracture repair by promoting bone regeneration through regulation of signal transmission among different cell types [38]. Zhang et al. enhanced NGF-TrkA signaling in bone defect areas by constructing engineered sensory nerve biomaterials, thereby significantly promoting bone defect repair [39]. In our previous study, we found that the lack of CGRP sensory nerve signaling was a key factor contributing to reduced healing capacity in osteoporotic fractures [8]. In this study, CGRP-functionalized MXene nanosheets were incorporated into the hydrogel, allowing the slow release of CGRP during the mid-to-late stages of fracture repair. During the critical stage of fracture remodeling, CGRP signaling regulated angiogenesis and callus mineralization, facilitating effective crosstalk between nerves, blood vessels, and bone, ultimately promoting fracture repair.

The degradation profile of the OCS-MPC hydrogel is critical to its therapeutic performance in osteoporotic fracture repair. This hydrogel undergoes enzymatic breakdown primarily through the hydrolysis of OHA-PBA and CMCS components, enabling a gradual and predictable degradation process. In vivo studies on similar hydrogel systems have reported degradation periods ranging from 2 to 4 weeks, which correspond well with the typical timeline of bone healing [40]. This controlled degradation ensures a sustained release of SDF-1 and CGRP, supporting bone regeneration while avoiding premature clearance of bioactive factors. Beyond bone regeneration, the dual-phase release strategy of OCS-MPC hydrogel holds promise for broader applications in regenerative medicine. Similar hydrogel systems have been explored for cartilage repair, nerve regeneration, and soft tissue engineering [29,41]. The ability to fine-tune the release kinetics of multiple bioactive factors makes this hydrogel a promising candidate for controlled tissue regeneration in various clinical settings. Future studies should explore its application in minimally invasive treatments and other orthopedic or tissue engineering scenarios.

Our work has some limitations. First, we developed the OCS-MPC hydrogel and focused on its effects on osteogenesis, angiogenesis, and nerve regeneration. However, we did not investigate the specific mechanism by which the OCS-MPC hydrogel facilitates crosstalk between nerves, blood vessels, and bone. This will be the focus of future studies. Second, the clinical application of nerve-vascular-bone interactions in osteoporotic fracture repair has not yet been evaluated.

## 4 Conclusion

In conclusion, we designed the OCS-MPC smart composite hydrogel and demonstrated its ability to effectively promote angiogenesis, osteogenic differentiation, and nerve regeneration in vitro. More importantly, the OCS-MPC smart composite hydrogel mimics the natural fracture healing process by regulating the sequential release of SDF-1/CGRP, promoting early callus formation during osteoporotic fracture repair, and accelerating callus mineralization and remodeling in later stages, ultimately enhancing fracture healing.

## 5 Experimental section

### 5.1 Chemicals

Ti_3_AlC_2_ (T302678), LiF (L434126), and HCl (H475775) were purchased from Aladdin Ltd. (China). Dopamine hydrochloride (D756911), sodium hyaluronate (HA, H909935), and carboxymethyl chitosan (CMCS, C804727) were obtained from Macklin Biochemical (China). Tris solution (ST788) and SDF-1 (P6749) were purchased from Beyotime Biotechnology (China). Sodium periodate (NaIO_4_, 83100A), ethylene glycol (C_2_H_6_O_2_, 13208A), and 4-aminobenzeneboronic acid hydrochloride (84253B) were obtained from Adamas-beta (China). N,N-Dimethylformamide (DMF, 76259B) was purchased from Greagent (China), and CGRP was obtained from MedChemExpress (HY-P0203, USA).

### 5.2 Synthesis of MXene@PDA/CGRP

Single-layer MXene was synthesized using the MILD method [42]. Ti_3_AlC_2_ (MAX) was added to an etching solution containing LiF (1 g) and 9 M HCl (20 mL) and reacted at 35 °C for 24 h. After etching, the product was washed with deionized water and centrifuged repeatedly until the pH of the supernatant exceeded 6. The residue was redispersed in deionized water, ultrasonicated for 60 min, and the dark green supernatant was collected by centrifugation, followed by freeze-drying to obtain single-layer Ti_3_C_2_ (MXene). Subsequently, 10 mg of dopamine was dissolved in 20 mL of Tris solution (10 mM, pH 8.5), and 6 mg of Ti_3_C_2_ MXene powder was added. After ultrasonication for 30 min, a uniform suspension was obtained. This suspension was stirred under dark conditions for 24 h, centrifuged at 12,000 rpm for 10 min, and washed three times with deionized water to obtain MXene@PDA precipitate, which was then freeze-dried to yield MXene@PDA nanosheets. Finally, 1.5 mg of MXene@PDA nanosheets were dissolved in 1.25 mL of an aqueous solution containing 10 μM CGRP and incubated overnight at 4 °C under dark conditions. The solution was centrifuged, washed, and freeze-stored to obtain MXene@PDA/CGRP for further applications.

### 5.3 Synthesis of OCS-MPC hydrogel

OHA was synthesized following previously reported methods [43]. Briefly, 2 g of HA was dissolved in 200 mL of deionized water and stirred until fully dissolved, followed by the addition of 1 g of sodium periodate. The reaction mixture was stirred at room temperature (25 °C) for 24 h in the dark, after which 2 mL of ethylene glycol was added and stirred for an additional hour to terminate the reaction. The product was dialyzed (MWCO 3500) and freeze-dried to yield OHA, which was stored at 4 °C for future use. The synthesis of OHA-PBA was performed as follows: 1 g of OHA was dissolved in 100 mL of a 1:1 mixture of deionized water and DMF, followed by the addition of 0.2 g of 4-aminobenzeneboronic acid hydrochloride. After dissolution, the pH was adjusted to 7 using 1 M NaOH, and the mixture was stirred at room temperature (25 °C) for 24 h. The product was then dialyzed and freeze-dried to obtain OHA-PBA. To prepare the OHA-PBA solution, 62.5 mg of OHA-PBA was dissolved in 1.25 mL of PBS (10 mM, pH 7.4), and 500 ng/mL SDF-1 was added and mixed thoroughly for later use. For the synthesis of the final OCS-MPC hydrogel, 62.5 mg of CMCS was dissolved in 1.25 mL of PBS (10 mM, pH 7.4) and mixed thoroughly, followed by the addition of 1.5 mg of MXene@PDA/CGRP. The mixture was shaken overnight. Finally, the OHA-PBA solution containing 500 ng/mL SDF-1 was mixed with the CMCS/MXene@PDA/CGRP mixture and incubated at 37 °C for 2 h to allow for complete gelation. The final composite hydrogel was then stabilized at 4 °C before further use.

### 5.4 Materials characterization

The morphology and surface features of MAX, single-layer MXene, and MXene@PDA nanosheets were characterized using scanning electron microscopy (SEM; Philips, Netherlands). Elemental surface distribution was analyzed using energy dispersive spectroscopy (EDS). Transmission electron microscopy (TEM; FEI, USA) was used to observe the nanomaterials' microstructure. Crystal structure analysis was performed using X-ray diffraction (XRD; Rigaku, Japan), and surface chemical states were analyzed using X-ray photoelectron spectroscopy (XPS; Thermo Fisher Scientific, UK). The morphology of the hydrogels was observed via SEM, and rheological properties were measured using a Haake rotational rheometer (Haake, Germany). The chemical structure of the hydrogels was analyzed using ^1^HNMR (Bruker, Germany).

### 5.5 Swelling and degradation studies

At 37 °C, the OHA-PBA/CMCS hydrogel was immersed in PBS, with the initial weight recorded as W_0_. After 24 h, the hydrogel was carefully removed, blotted with filter paper to remove surface moisture, and weighed (W_t_). The equilibrium swelling ratio (ESR) was calculated using the formula: ESR = (W_t_/W_0_) × 100 %. For the in vitro degradation study, the hydrogel was immersed in PBS and incubated at 37 °C with shaking at 100 rpm. The initial weight of the hydrogel was recorded as W_0_, and PBS was replaced daily. At predetermined time points, samples were removed, blotted to remove surface moisture, and weighed (W_t_). The degradation percentage was calculated as: weight loss rate = [(W_0_ − W_t_)/W_0_] × 100 %.

### 5.6 In vitro release of CGRP and SDF-1

A 100 μL sample of OCS-MPC hydrogel was immersed in 3 mL of PBS and incubated in a shaking incubator at 37 °C and 100 rpm. At predetermined time points, 1 mL of the supernatant was collected and replaced with 1 mL of fresh PBS. The concentrations of CGRP (CSB-EQ (0277)06MO, CUSABIO, China) and SDF-1(CSB-EQ (0274)94MO, CUSABIO, China) released were quantified using enzyme-linked immunosorbent assay (ELISA).

### 5.7 Testing of rapid gelation, injectability, and self-healing properties of OCS-MPC hydrogel

To evaluate the rapid gelation and injectability of the OCS-MPC hydrogel, an inclined vial test and an injection experiment were conducted. Equal volumes of 5 % carboxymethyl chitosan (CMCS) solution and 5 % oxidized hyaluronic acid-PBA (OHA-PBA) solution were mixed and thoroughly stirred to obtain a homogeneous mixture. The mixture was placed at 37 °C, and the sol-gel transition time was recorded using the inclined vial method to determine when the liquid lost its fluidity. To assess injectability, the mixed solution was injected through a 1 mL syringe into a petri dish to simulate filling a bone defect site. After injection, the hydrogel quickly solidified and was able to form target structures, such as the word “BONE,” demonstrating its excellent shape retention ability. For self-healing evaluation, the prepared OCS-MPC hydrogel was cut, and the cut ends were brought into contact. The hydrogel's ability to reattach and heal was observed after allowing it to stand for a period of time.

### 5.8 Evaluation of In vitro biocompatibility and angiogenesis of OCS-MPC hydrogel

#### 5.8.1 Preparation of hydrogel extract solution

OC-MP and OCS-MPC hydrogels were immersed in 10 mL of DMEM (11965092, Gibco, USA) culture medium and incubated at 37 °C for 48 h to allow thorough soaking. The supernatant was then collected and filtered to remove bacteria, viruses, and other pyrogens. The extract was supplemented with 10 % fetal bovine serum (10099141C, Gibco, USA) and 1 % penicillin-streptomycin (15140122, Gibco, USA) for use in subsequent experiments.

#### 5.8.2 Cell Culture and viability assay

HUVECs were seeded into 96-well plates at a density of 5 × 10^3^ cells per well and cultured at 37 °C with 5 % CO_2_. After cell attachment, the culture medium was replaced with hydrogel extract, while the control group received complete DMEM. After 3 days of incubation, the medium was aspirated, cells were washed with PBS, and a Calcein-AM/PI staining kit (C2015M, Beyotime, China) was used to assess cell viability. For the cytotoxicity assessment of cell proliferation, HUVECs were seeded into 96-well plates at a density of 5 × 10^3^ cells per well and cultured at 37 °C with 5 % CO_2_. After cell attachment, the culture medium was replaced with hydrogel extract, while the control group received complete DMEM. Cell proliferation was evaluated at predetermined time points (1, 4, and 7 days) using the CCK-8 assay kit (C0038, Beyotime, China), and the absorbance was measured at 450 nm using a microplate reader.

#### 5.8.3 In vitro angiogenesis evaluation

HUVECs were seeded in 6-well plates, and upon reaching 90 % confluency, a scratch assay was performed using a 200 μL pipette tip. Cells were washed with sterile PBS to remove debris, and the medium was replaced with hydrogel extract containing 2 % fetal bovine serum. The control group received DMEM containing 2 % fetal bovine serum. Images were taken at 0, 12, and 24 h, and the scratch area was analyzed using ImageJ software. To simulate the chemotactic migration of HUVECs in vitro, 2 × 10^4^ HUVECs were seeded in the upper chamber of a Transwell (353097, BD Falcon, USA), while the lower chamber was filled with hydrogel extract. The control group used complete DMEM. After 24 h of culture, cells were fixed with paraformaldehyde, stained with crystal violet, and cell migration was observed under a microscope. To assess tube formation, 1.2 × 10^5^ HUVECs were mixed with hydrogel extract and seeded into a Matrigel-coated 48-well plate (354234, Corning, NY, USA). The cells were incubated for 6 h, and tube formation ability was evaluated under a microscope. The wound healing area was quantified using ImageJ by measuring the percentage of wound closure after 24 h relative to the initial scratch width. For tube formation analysis, total tube length and number of branches were quantified using ImageJ Fiji (v1.54f, NIH, Bethesda, USA).

#### 5.8.4 mRNA detection of angiogenic markers

HUVECs (4 × 10^5^ cells) were seeded in 6-well plates, and after cell adhesion, the medium was replaced with hydrogel extract. After 48 h of incubation, total RNA was extracted using an RNA extraction and purification kit (B0004DP, EZBioscience, USA). cDNA was synthesized using a cDNA reverse transcription kit (A0010CGQ, EZBioscience, USA). PCR analysis was carried out using Applied Biosystems QuantStudioTM 5 Real-Time PCR system (Applied Biosystems, Waltham, USA) and SYBR Green I Master Mix (A0012-R2, EZBioscience, USA). Primer sequences for target genes (VEGF, CD31, and HIF-1α) are listed in Table 1 (Sangon Biotech, China), with β-actin used as the housekeeping gene.

**Table 1:** Sequences of oligonucleotide primers for RT-qPCR.

Gene	Forward primer (5′-3′)	Reverse primer (5′-3′)
CD31	CTGGCCCAGGAGTTTCCAGA	GTTGCCACTGTGCTCCACCA
HIF1-α	AGAGGTTGAGGGACGGAGAT	GACGTTCAGAACTTATCCTACCAT
VEGF	AGAAGGAGGAGGGCAGAATCATCAC	GGGCACACAGGATGGCTTGAAG
β-actin-H	ACAGAGCCTCGCCTTTGC	CCACCATCACGCCCTGG
COL-1	CGACCTCAAGATGTGCCACT	CCATCGGTCATGCTCTCTCC
OCN	AGCAGCTTGGCCCAGACCTA	TAGCGCCGGAGTCTGTTCACTAC
OPN	CAGCAGCTCACACTGAAGAAGC	AGAATTCAGCCAGGAGAACTGC
β-actin-M	AGATGTGGATCAGCAAGCAG	GCGCAAGTTAGGTTTTGTCA

#### 5.8.5 Detection of angiogenic proteins

HUVECs (4 × 10^5^ cells) were seeded in 6-well plates, and after cell adhesion, the medium was replaced with hydrogel extract. After 48 h, total protein was extracted from each group of cells using RIPA lysis buffer (PC101, EpiZyme, China), and protein concentrations were determined using a BCA protein assay kit (23227, Thermo Scientific, UK). Extracted proteins were separated by SDS-PAGE and transferred onto a PVDF membrane. The membrane was blocked with 5 % skim milk for 1 h to prevent nonspecific binding, washed three times with TPBS, and incubated overnight at 4 °C with primary antibodies against VEGF (1:2000, 19003-1-AP, Proteintech, China), CD31 (1:2000, 11265-1-AP, Proteintech, China), and HIF-1α (1:2000, 20960-1-AP, Proteintech, China). The membrane was then incubated with the appropriate secondary antibody at room temperature for 1 h. Finally, protein bands were visualized using the FluorChem imaging system (Tanon, Shanghai, China).

### 5.9 In Vitro Osteogenic evaluation of OCS-MPC hydrogel

#### 5.9.1 Extraction and culture of bone marrow-derived mesenchymal stem cells

Bone marrow-derived mesenchymal stem cells (mBMSCs) were isolated from 3-week-old male C57BL/6J mice. The bone marrow suspension was seeded in culture flasks containing α-MEM (12571063, Gibco, USA) supplemented with 10 % fetal bovine serum. Cells were cultured at 37 °C in a 5 % CO_2_ atmosphere, and passages 3 to 5 were used for subsequent experiments.

#### 5.9.2 In Vitro Osteogenic differentiation assay

mBMSCs (5 × 10^4^ cells per well) were co-cultured in 24-well plates with osteogenic induction medium containing hydrogel extract for 7 and 14 days. Cells were washed with PBS, fixed with 4 % paraformaldehyde for 30 min, and stained using an ALP staining kit (C3206, Beyotime, China). Positive staining areas were quantified using ImageJ software. For mineralization assessment, mBMSCs (5 × 10^4^ cells per well) were co-cultured in 24-well plates with osteogenic induction medium containing hydrogel extract for 21 and 28 days. Cells were washed with PBS, fixed with 4 % paraformaldehyde for 30 min, and stained with 1 % Alizarin Red S (R30132, Shyuanye, China) for 30 min. Residual stain was washed off with deionized water, and stained cells were observed and imaged under an optical microscope. Quantification of stained areas was performed using ImageJ software.

#### 5.9.3 mRNA detection of osteogenic markers

mBMSCs (4 × 10^5^ cells) were seeded in 6-well plates, and after cell adhesion, the medium was replaced with osteogenic induction medium containing hydrogel extract and cultured for 7 days. mRNA extraction and PCR analysis were performed as previously described. Primer sequences for the target genes (OCN, COL-1, and OPN) are listed in Table 1 (Sangon Biotech, China), with β-actin used as the housekeeping gene.

#### 5.9.4 Detection of osteogenic proteins

mBMSCs (4 × 10^5^ cells) were seeded in 6-well plates, and after cell adhesion, the medium was replaced with osteogenic induction medium containing hydrogel extract and cultured for 21 days. Protein extraction was conducted as previously described, and Western blot analysis was performed to detect the expression of osteogenic markers: OCN (1:1000, A6205, ABclonal, China), COL-1 (1:2000, 66761-1-Ig, Proteintech, China), and OPN (1:1000, 22952-1-AP, Proteintech, China).

### 5.10 In vitro evaluation of OCS-MPC hydrogel in promoting sensory neuron differentiation

#### 5.10.1 Extraction of dorsal root ganglion cells

Dorsal root ganglion cells (DRGs) were extracted from 4-week-old mice using an adapted and improved version of established methods [44,45]. Euthanized mice were disinfected with 70 % ethanol to prevent contamination from fur. The head was removed as close to the base of the skull as possible to avoid damaging the cervical vertebrae. The skin was cut open upward from the pelvis, and the dorsal skin was removed. The spine was cut from the neck to the base of the tail and separated from surrounding soft tissues, with muscle and fat tissues removed. The spine was bisected, and the spinal cord was carefully stripped away. Collected DRGs were placed in pre-cooled HBSS and centrifuged to obtain a pellet. DRGs were digested at 37 °C with type II collagenase (17101015, Gibco, USA) for 60 min, followed by an additional 30-min digestion with 1 mL of 0.25 % trypsin (25200072,Gibco, USA). The supernatant was removed by centrifugation, and DRGs were resuspended in specialized DRG culture medium before being seeded in poly-D-lysine (PDL)-coated 24-well plates for culture. After two days of incubation, the culture medium was replaced with nerve induction medium containing hydrogel extract (supplemented with 2 % B-27 and 2 mM GlutaMAX™, both from Gibco, 17504044, 35050061) for further culture.

#### 5.10.2 Evaluation of DRG differentiation

Western blot and immunofluorescence analyses were performed after 48 h of treatment for protein expression analysis, and after 7–10 days of induction for neurite growth assays. After treatment with OCS-MPC for one week, DRGs were washed with PBS, fixed in 4 % paraformaldehyde for 15 min, and permeabilized with 0.1 % Triton X-100 for 10 min. Samples were then blocked at room temperature with 3 % bovine serum albumin (A8020, Solarbio, China) for 1 h. The blocked samples were incubated overnight at 4 °C with primary antibodies against β3-tubulin (1:200, 5568, CST, USA) and CGRP (1:200, 14959, CST, USA).

Subsequently, the samples were incubated with Cy3-conjugated goat anti-rabbit IgG (H + L) secondary antibody (1:500, A0516, Beyotime, China) at room temperature for 1 h. DRGs nuclei were then stained with DAPI (C1006, Beyotime, China), and fluorescent images were captured using a fluorescence microscope (Nikon, Japan). Fluorescence intensity was quantified using ImageJ software.

### 5.11 In vivo evaluation of OCS-MPC hydrogel in accelerating osteoporotic fracture repair

#### 5.11.1 Animal model establishment

To investigate the effects of OCS-MPC hydrogel on osteoporotic fractures, an animal model of osteoporosis and fracture was established. Ovariectomized (OVX) mice were selected as the model due to their well-documented ability to mimic postmenopausal osteoporosis in humans, characterized by estrogen deficiency-induced bone loss and impaired fracture healing. Ten-week-old female C57 mice underwent bilateral ovariectomy to induce osteoporosis. Specifically, after intraperitoneal anesthesia with 0.5 % pentobarbital, a small incision was made bilaterally in the abdomen to access and remove the ovaries. In the sham group, only the fat tissue around the ovary was removed. Bone density was assessed by micro-CT scanning two months post-surgery to confirm successful establishment of the osteoporosis model. A fracture model was subsequently established. Under anesthesia, mice were fixed on the surgical table, and the left hind limb was disinfected with povidone-iodine. A 1 cm skin incision was made proximally to the distal femur to expose the femur through blunt dissection. A 0.5 mm Kirschner wire was retrogradely inserted into the femoral condyle's medullary cavity, and an osteotomy was performed at the midshaft to create a stable fracture. The OVX model recapitulates the pathophysiological changes observed in postmenopausal osteoporosis, including decreased bone mass, trabecular microarchitecture deterioration, and impaired bone healing. This model is widely accepted for preclinical evaluation of bone regeneration strategies. OCS-MPC hydrogel or control OC-MP hydrogel was then injected into the fracture site, and the incision was closed layer by layer. Postoperative care included daily wound disinfection and intramuscular injection of antibiotics for three days. At 1-, 4-, and 8-weeks post-surgery, six mice per group were euthanized for sample collection and assessment of fracture healing. The study was approved by the Ethics Committee of Tongren Hospital, affiliated with Shanghai Jiao Tong University School of Medicine (ethical approval number A2023-057-01). All experiments were conducted in accordance with the guidelines of the Experimental Animal Science at Shanghai Jiao Tong University School of Medicine.

#### 5.11.2 Micro-CT Scanning and analysis

The femoral samples were scanned using a Skyscan1276 micro-computed tomography (μCT) scanner. For stabilization during scanning, femoral specimens were fixed in a specialized container. Each femur was scanned at 100 kV and 200 μA, with a resolution of 6 μm. Scanned data were reconstructed using NRecon software, and quantitative analysis of the volume of interest (VOI) was conducted using Bruker's CTAN software. The VOI encompassed the entire healing tissue, excluding the original bone. The primary parameters analyzed included bone volume to total volume ratio (BV/TV), trabecular thickness (Tb.Th), and trabecular number (Tb.N). All analyses were performed in a blinded manner to ensure objectivity and accuracy of the data.

#### 5.11.3 Histological evaluation

To further assess fracture site repair, mice were euthanized, and cardiac perfusion was performed with saline followed by 4 % paraformaldehyde (PFA). Femoral samples were fixed in 4 % PFA for 48 h and subsequently decalcified in 10 % EDTA solution for 4 weeks. After decalcification, the samples were embedded in paraffin, and sections were cut at a thickness of 5 μm. The sections were stained with hematoxylin and eosin (HE), Masson's trichrome, and Safranin O/Fast Green (SOFG) to analyze callus growth and remodeling.

#### 5.11.4 Immunofluorescence and immunohistochemistry

Paraffin sections were deparaffinized in xylene and dehydrated through a graded ethanol series (100 %, 95 %, 80 %, and 75 %). Antigen retrieval was performed using citrate buffer with microwave heating. After cooling, sections were blocked with 3 % bovine serum albumin (BSA) to prevent nonspecific binding. Sections were incubated with diluted primary antibodies at 4 °C for 12 h. After washing, secondary antibodies were applied, and the sections were incubated at room temperature for 60 min. Finally, cell nuclei were stained with DAPI, and imaging was performed using an Olympus microscope. Quantitative analysis of positive cells or marked areas was conducted using ImageJ software.

### 5.12 Statistical analysis

All data were analyzed using GraphPad Prism 9.0 software and are presented as mean ± standard deviation (mean ± SD). One-way analysis of variance (ANOVA) was conducted to compare different experimental groups, while an independent sample *t*-test was used for comparisons between two groups. The significance level of p < 0.05 was considered statistically significant. Each experiment was repeated at least three times to ensure the reliability and reproducibility of the results. All analyses were conducted in a double-blind manner to minimize potential bias.

## CRediT authorship contribution statement

**Yuan Wang:** Writing – review & editing, Writing – original draft, Visualization, Supervision, Software, Resources, Project administration, Methodology, Funding acquisition, Formal analysis, Conceptualization. **Zhen Pan:** Writing – original draft, Visualization, Methodology, Investigation. **Qianliang Wang:** Methodology, Project administration, Validation, Writing – original draft. **Yuexia Shu:** Writing – original draft, Visualization, Software, Methodology. **Zhenyu Tan:** Resources, Methodology. **Yujie Chen:** Supervision, Resources. **Jieming He:** Resources. **Jia Wang:** Resources. **Jielin Wang:** Writing – original draft, Supervision, Resources, Project administration, Conceptualization. **Jun Yan:** Supervision, Resources, Conceptualization.

## Declaration of competing interest

The authors declare that there are no known conflicts of interest associated with this publication, and there has been no significant financial support that could have influenced its outcome. All funding sources have been acknowledged appropriately in the manuscript.

The authors confirm that the work presented in this manuscript is original and has not been previously published, nor is it currently being considered for publication elsewhere. The authors also confirm that there are no relationships or activities that could inappropriately influence or bias the content of the manuscript.

## Data Availability

Data will be made available on request.

## Appendix A Supplementary data

Figure S1. Characterization and Properties of OCS-MPC Hydrogel (A) High-resolution XPS spectra of C 1s for MXene and C 1s, N 1s for MXene@PDA. (B-C) Chemical structures and synthesis schematics of OHA-PBA and OHA-PBA/ CMCS. (D-E) Representative live/dead staining images and quantitative analysis of HUVECs treated with OC-MP and OCS-MPC. Scale bar = 200 μm. n = 3. All data are representative of at least three independent experiments. Data are presented as mean ± SEM. ∗P < 0.05, ∗∗P < 0.01, ∗∗∗P < 0.001, ∗∗∗P < 0.0001. Figure S2. In Vitro Osteogenic, Angiogenic, and Neurogenic Properties of OCS-MPC (A) qPCR analysis of osteogenic-related mRNA (OCN, COL-1, and OPN) expression in BMSCs cultured with OC-MP and OCS-MPC (n = 3). (B-C) Representative Transwell migration assay images and quantitative analysis of HUVECs treated with OC-MP and OCS-MPC for 24 h (scale bar = 200 μm, n = 3). (D) Quantitative analysis of tube formation assay of HUVECs treated with OC-MP and OCS-MPC (n = 3). (E) qPCR analysis of angiogenesis-related mRNA (VEGF, CD31, and HIF-1α) expression in HUVECs cultured with OC-MP and OCS-MPC (n = 3). All data are representative of at least three independent experiments. Data are presented as mean ± SEM. ∗P < 0.05, ∗∗P < 0.01, ∗∗∗P < 0.001, ∗∗∗∗P < 0.0001. Figure S3. OCS-MPC Promotes Callus Formation and Remodeling in Osteoporotic Fracture Healing (A) Representative micro-CT images of femoral fractures in mice treated with OC-MP or OCS-MPC at 1-week post-fracture. Scale bar = 1 mm. n = 6. (B) Representative Masson-stained images of femoral fractures in WT mice treated with OC-MP or OCS-MPC at 1-week post-fracture. Upper panels show global views; lower panels show close-ups of the fracture sites. Scale bar = 1 mm. (C) Immunohistochemical staining images of Col-2 protein levels in calluses from mice treated with OC-MP or OCS-MPC at 1-week post-fracture. Scale bar = 1 mm. (D) Representative micro-CT images of femoral fractures in mice treated with OC-MP or OCS-MPC at 4 weeks post-fracture. Scale bar = 1 mm. n = 6. (E) Representative Masson-stained images of femoral fractures in WT mice treated with OC-MP or OCS-MPC at 4 weeks post-fracture. Upper panels show global views; lower panels show close-ups of the fracture sites. Scale bar = 1 mm. n = 3 per group. All data are representative of at least three independent experiments. Data are presented as mean ± SEM. ∗P < 0.05, ∗∗P < 0.01, ∗∗P < 0.001, ∗∗∗∗P < 0.0001.Multimedia component 1

## References

[1] Cauley J.A. (2017). Osteoporosis: fracture epidemiology update 2016. Curr. Opin. Rheumatol..

[2] Khosla S., Farr J.N., Monroe D.G. (2022). Cellular senescence and the skeleton: pathophysiology and therapeutic implications. J. Clin. Investig..

[3] Nakahama K. (2010). Cellular communications in bone homeostasis and repair. Cell. Mol. Life Sci..

[4] Madl C.M., Heilshorn S.C., Blau H.M. (2018). Bioengineering strategies to accelerate stem cell therapeutics. Nature.

[5] Li J., Zhang Z., Tang J., Hou Z., Li L., Li B. (2024). Emerging roles of nerve-bone axis in modulating skeletal system. Med. Res. Rev..

[6] Nazzal M.K., Morris A.J., Parker R.S., White F.A., Natoli R.M., Kacena M.A., Fehrenbacher J.C. (2024). Do not lose your nerve, Be callus: insights into neural regulation of fracture healing. Curr. Osteoporos. Rep..

[7] Li Z., Meyers C.A., Chang L., Lee S., Li Z., Tomlinson R., Hoke A., Clemens T.L., James A.W. (2019). Fracture repair requires TrkA signaling by skeletal sensory nerves. J. Clin. Investig..

[8] Shu Y., Tan Z., Pan Z., Chen Y., Wang J., He J., Wang J., Wang Y. (2024). Inhibition of inflammatory osteoclasts accelerates callus remodeling in osteoporotic fractures by enhancing CGRP+TrkA+ signaling. Cell Death Differ..

[9] Wang Q., Qin H., Deng J., Xu H., Liu S., Weng J., Zeng H. (2023). Research progress in calcitonin gene-related peptide and bone repair. Biomolecules.

[10] Wang F., Deng Y., Wang J., Yu L., Ding F., Lian W., Liu Q., Lin X. (2021). The PLGA nanoparticles for sustainable release of CGRP to ameliorate the inflammatory and vascular disorders in the lung of CGRP-deficient rats. Drug Deliv..

[11] Shang F., Yu Y., Liu S., Ming L., Zhang Y., Zhou Z., Zhao J., Jin Y. (2021). Advancing application of mesenchymal stem cell-based bone tissue regeneration. Bioact. Mater..

[12] Bogdanowicz D.R., Lu H.H. (2017). Designing the stem cell microenvironment for guided connective tissue regeneration. Ann. N. Y. Acad. Sci..

[13] Hall B.K., Miyake T. (2000). All for one and one for all: condensations and the initiation of skeletal development. Bioessays.

[14] Cui Z.-K., Kim S., Baljon J.J., Wu B.M., Aghaloo T., Lee M. (2019). Microporous methacrylated glycol chitosan-montmorillonite nanocomposite hydrogel for bone tissue engineering. Nat. Commun..

[15] Chaudhuri O., Gu L., Klumpers D., Darnell M., Bencherif S.A., Weaver J.C., Huebsch N., Lee H.-P., Lippens E., Duda G.N., Mooney D.J. (2016). Hydrogels with tunable stress relaxation regulate stem cell fate and activity. Nat. Mater..

[16] Struthers A.D., Brown M.J., Macdonald D.W.R., Beacham J.L., Stevenson J.C., Morris H.R., Macintyre I. (1986). Human calcitonin gene related peptide: a potent endogenous vasodilator in man. Clin. Sci. (Lond.).

[17] Naguib M., Kurtoglu M., Presser V., Lu J., Niu J., Heon M., Hultman L., Gogotsi Y., Barsoum M.W. (2011). Two-dimensional nanocrystals produced by exfoliation of Ti3 AlC2. Adv. Mater..

[18] Ren T., Dailey H.L. (2020). Mechanoregulation modeling of bone healing in realistic fracture geometries. Biomech. Model. Mechanobiol..

[19] Clynes M.A., Harvey N.C., Curtis E.M., Fuggle N.R., Dennison E.M., Cooper C. (2020). The epidemiology of osteoporosis. Br. Med. Bull..

[20] Xie Y., Zhang L., Xiong Q., Gao Y., Ge W., Tang P. (2019). Bench-to-bedside strategies for osteoporotic fracture: from osteoimmunology to mechanosensation. Bone Res.

[21] Zhai P., Peng X., Li B., Liu Y., Sun H., Li X. (2020). The application of hyaluronic acid in bone regeneration. Int. J. Biol. Macromol..

[22] Carboxymethyl chitosan: Properties and biomedical applications - PubMed, (n.d.). https://pubmed.ncbi.nlm.nih.gov/30267813/(accessed October 14, 2024).

[23] Geng Y., Xue H., Zhang Z., Panayi A.C., Knoedler S., Zhou W., Mi B., Liu G. (2023). Recent advances in carboxymethyl chitosan-based materials for biomedical applications. Carbohydr. Polym..

[24] Li L., Wang N., Jin X., Deng R., Nie S., Sun L., Wu Q., Wei Y., Gong C. (2014). Biodegradable and injectable in situ cross-linking chitosan-hyaluronic acid based hydrogels for postoperative adhesion prevention. Biomaterials.

[25] Wang S., Chi J., Jiang Z., Hu H., Yang C., Liu W., Han B. (2021). A self-healing and injectable hydrogel based on water-soluble chitosan and hyaluronic acid for vitreous substitute. Carbohydr. Polym..

[26] Yue Y., Liu Y., Lin Y., Guo F., Cai K., Chen S., Zhang W., Tang S. (2024). A carboxymethyl chitosan/oxidized hyaluronic acid composite hydrogel dressing loading with stem cell exosome for chronic inflammation wounds healing. Int. J. Biol. Macromol..

[27] Zhang F., Zhang S., Cui S., Jing X., Feng Y., Coseri S. (2024). Rapid self-healing carboxymethyl chitosan/hyaluronic acid hydrogels with injectable ability for drug delivery. Carbohydr. Polym..

[28] Pan S., Yin J., Yu L., Zhang C., Zhu Y., Gao Y., Chen Y. (2020). 2D MXene‐integrated 3D‐printing scaffolds for augmented osteosarcoma phototherapy and accelerated tissue reconstruction. Adv. Sci..

[29] Lv Z., Hu T., Bian Y., Wang G., Wu Z., Li H., Liu X., Yang S., Tan C., Liang R., Weng X. (2023). A MgFe-LDH nanosheet-incorporated smart thermo-responsive hydrogel with controllable growth factor releasing capability for bone regeneration. Adv. Mater..

[30] Lin H., Sohn J., Shen H., Langhans M.T., Tuan R.S. (2019). Bone marrow mesenchymal stem cells: aging and tissue engineering applications to enhance bone healing. Biomaterials.

[31] (2014). Stem cell aging: mechanisms, regulators and therapeutic opportunities -. PubMed.

[32] Lee J.H., Jung H.K., Han Y.-S., Yoon Y.M., Yun C.W., Sun H.Y., Cho H.W., Lee S.H. (2016). Antioxidant effects of Cirsium setidens extract on oxidative stress in human mesenchymal stem cells. Mol. Med. Rep..

[33] Gu C., Zhou Q., Hu X., Ge X., Hou M., Wang W., Liu H., Shi Q., Xu Y., Zhu X., Yang H., Chen X., Liu T., He F. (2024). Melatonin rescues the mitochondrial function of bone marrow‐derived mesenchymal stem cells and improves the repair of osteoporotic bone defect in ovariectomized rats. J. Pineal Res..

[34] Stromal Derived Factor-1/CXCR4 Axis Involved in Bone Marrow Mesenchymal Stem Cells Recruitment to Injured Liver - PubMed, (n.d.). https://pubmed.ncbi.nlm.nih.gov/26880995/(accessed October 14, 2024). 10.1155/2016/8906945 PMC4737461 26880995

[35] Wang Y., Sun J., Zhang Y., Liu W., Yang S., Yang J. (2021). *Stichopus japonicus* polysaccharide stimulates osteoblast differentiation through activation of the bone morphogenetic protein pathway in MC3T3-E1 cells. J. Agric. Food Chem..

[36] Niedermair T., Schirner S., Lasheras M.G., Straub R.H., Grässel S. (2020). Absence of α-calcitonin gene-related peptide modulates bone remodeling properties of murine osteoblasts and osteoclasts in an age-dependent way. Mech. Ageing Dev..

[37] Saul D., Khosla S. (2022). Fracture healing in the setting of endocrine diseases, aging, and cellular senescence. Endocr. Rev..

[38] Gao X., Zhang D., Xu C., Li H., Caron K.M., Frenette P.S. (2021). Nociceptive nerves regulate haematopoietic stem cell mobilization. Nature.

[39] Zhang Z., Wang F., Huang X., Sun H., Xu J., Qu H., Yan X., Shi W., Teng W., Jin X., Shao Z., Zhang Y., Zhao S., Wu Y., Ye Z., Yu X. (2023). Engineered sensory nerve guides self‐adaptive bone healing via NGF‐TrkA signaling pathway. Adv. Sci. (Weinh.).

[40] Hu B., Gao M., Boakye-Yiadom K.O., Ho W., Yu W., Xu X., Zhang X.-Q. (2021). An intrinsically bioactive hydrogel with on-demand drug release behaviors for diabetic wound healing. Bioact. Mater..

[41] Yang Z., Fu X., Ma D., Wang Y., Peng L., Shi J., Sun J., Gan X., Deng Y., Yang W. (2021). Growth factor-decorated Ti3 C2 MXene/MoS2 2D bio-heterojunctions with quad-channel photonic disinfection for effective regeneration of bacteria-invaded cutaneous tissue. Small.

[42] Merino S., Martín C., Kostarelos K., Prato M., Vázquez E. (2015). Nanocomposite hydrogels: 3D polymer-nanoparticle synergies for on-demand drug delivery. ACS Nano.

[43] Tan H., Chu C.R., Payne K.A., Marra K.G. (2009). Injectable in situ forming biodegradable chitosan–hyaluronic acid based hydrogels for cartilage tissue engineering. Biomaterials.

[44] Heinrich T., Hübner C., Kurth I. (2016). Isolation and primary cell culture of mouse dorsal root ganglion neurons. BIO-PROTOCOL.

[45] Smith P.R., Meyer A., Loerch S., Campbell Z.T. (2023). Protocol for the isolation and culture of mouse dorsal root ganglion neurons for imaging applications. STAR Protocols.

