# Supplementary figures and images for: Sequential SDF-1/CGRP-releasing smart composite hydrogel promotes osteoporotic fracture healing by targeting sensory nerve-regulated bone remodeling
Source: Mater Today Bio. 2025 Apr 17;32:101750. doi: 10.1016/j.mtbio.2025.101750 (PMC12054128; doi:10.1016/j.mtbio.2025.101750)

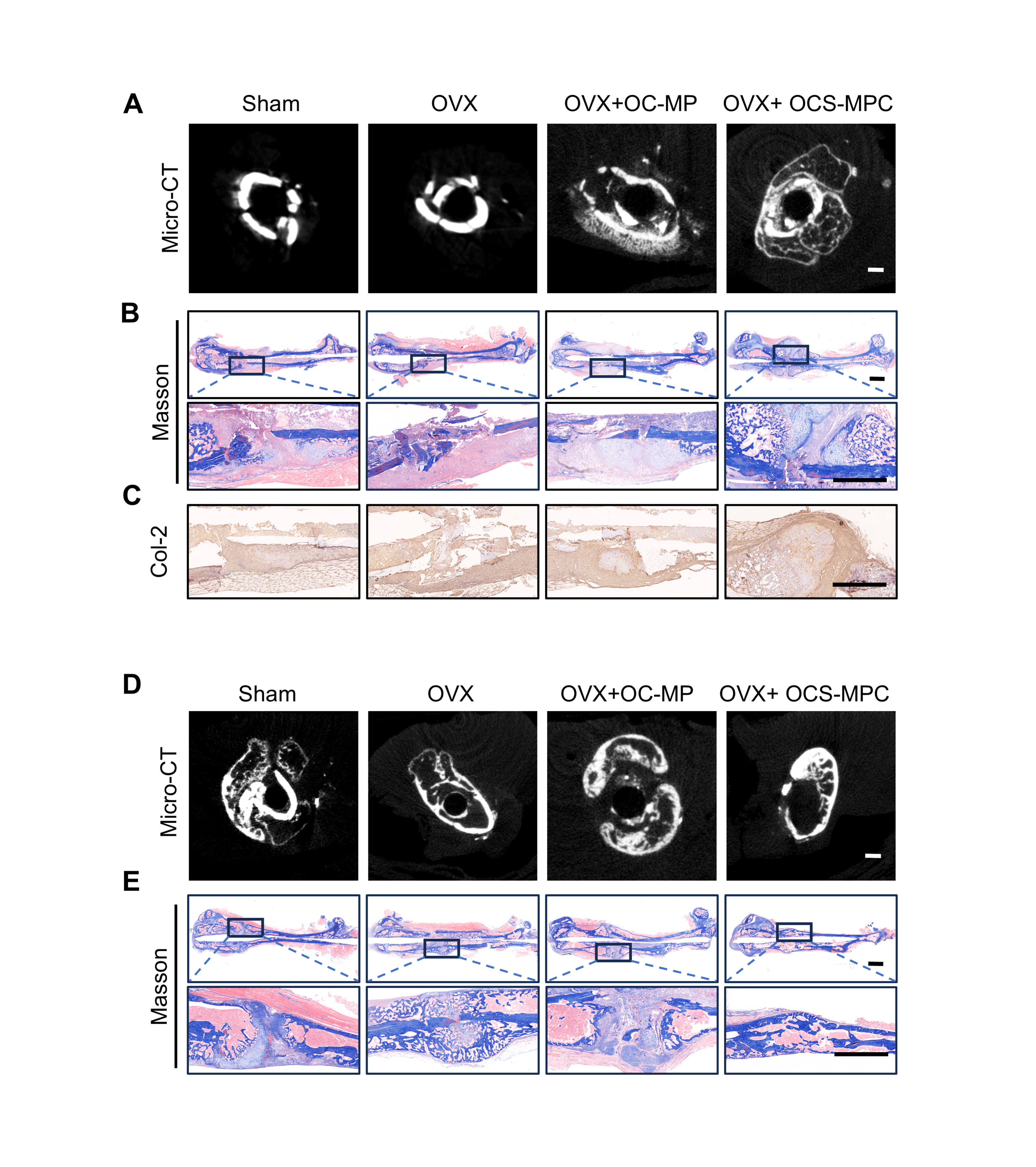

Supplement: Supplementary file 1 — Figure S1. Characterization and Properties of OCS-MPC Hydrogel (A) High-resolution XPS spectra of C 1s for MXene and C 1s, N 1s for MXene@PDA. (B-C) Chemical structures and synthesis schematics of OHA-PBA and OHA-PBA/ CMCS. (D-E) Representative live/dead staining images and quantitative analysis of HUVECs treated with OC-MP and OCS-MPC. Scale bar = 200 μm. n = 3. All data are representative of at least three independent experiments. Data are presented as mean ± SEM. ∗P < 0.05, ∗∗P < 0.01, ∗∗∗P < 0.001, ∗∗∗P < 0.0001. Figure S2. In Vitro Osteogenic, Angiogenic, and Neurogenic Properties of OCS-MPC (A) qPCR analysis of osteogenic-related mRNA (OCN, COL-1, and OPN) expression in BMSCs cultured with OC-MP and OCS-MPC (n = 3). (B-C) Representative Transwell migration assay images and quantitative analysis of HUVECs treated with OC-MP and OCS-MPC for 24 h (scale bar = 200 μm, n = 3). (D) Quantitative analysis of tube formation assay of HUVECs treated with OC-MP and OCS-MPC (n = 3). (E) qPCR analysis of angiogenesis-related mRNA (VEGF, CD31, and HIF-1α) expression in HUVECs cultured with OC-MP and OCS-MPC (n = 3). All data are representative of at least three independent experiments. Data are presented as mean ± SEM. ∗P < 0.05, ∗∗P < 0.01, ∗∗∗P < 0.001, ∗∗∗∗P < 0.0001. Figure S3. OCS-MPC Promotes Callus Formation and Remodeling in Osteoporotic Fracture Healing (A) Representative micro-CT images of femoral fractures in mice treated with OC-MP or OCS-MPC at 1-week post-fracture. Scale bar = 1 mm. n = 6. (B) Representative Masson-stained images of femoral fractures in WT mice treated with OC-MP or OCS-MPC at 1-week post-fracture. Upper panels show global views; lower panels show close-ups of the fracture sites. Scale bar = 1 mm. (C) Immunohistochemical staining images of Col-2 protein levels in calluses from mice treated with OC-MP or OCS-MPC at 1-week post-fracture. Scale bar = 1 mm. (D) Representative micro-CT images of femoral fractures in mice treated with OC-MP or OCS-MP [file mmc1.zip › Figure s3.tif]

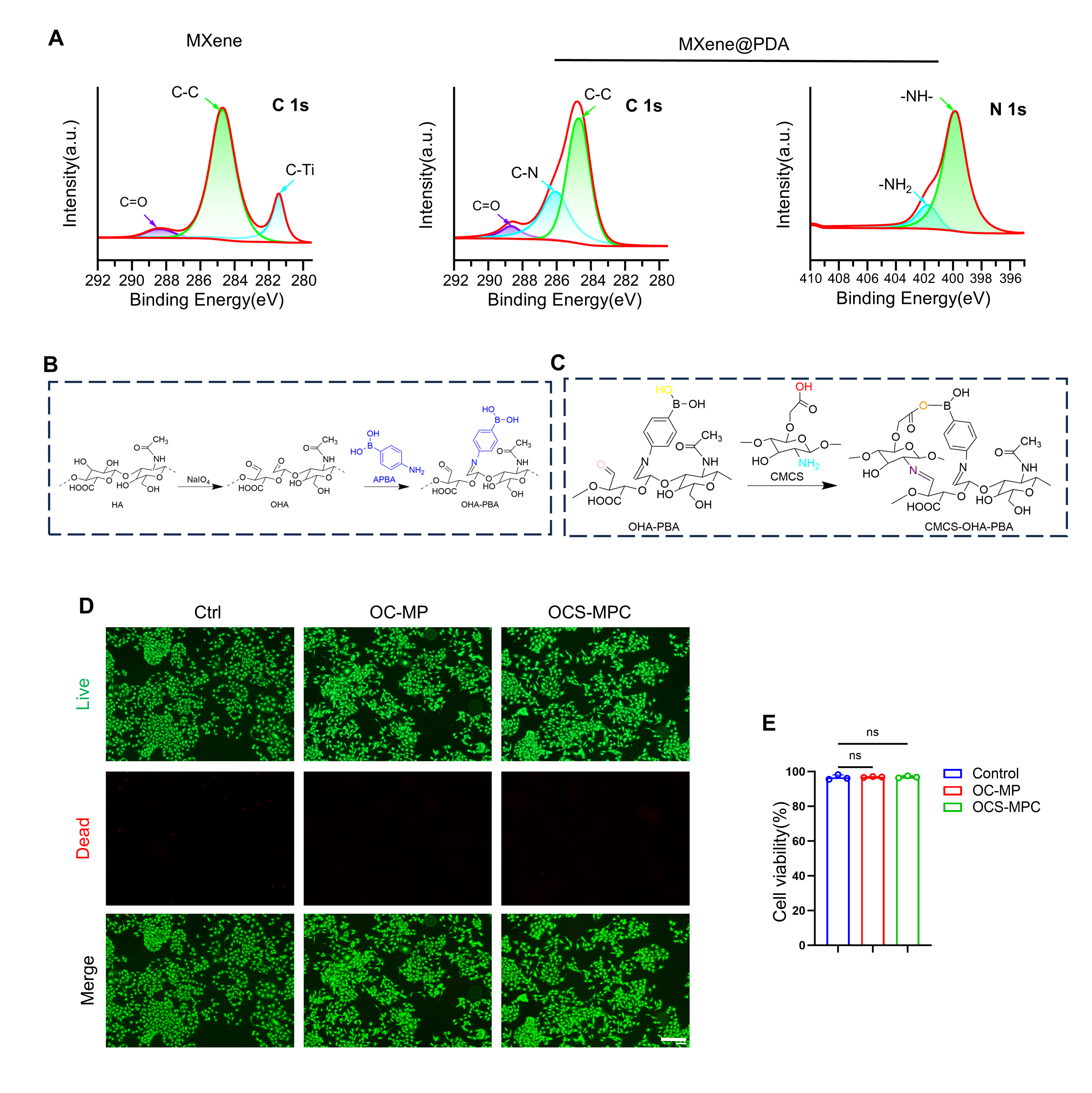

Supplement: Supplementary file 1 — Figure S1. Characterization and Properties of OCS-MPC Hydrogel (A) High-resolution XPS spectra of C 1s for MXene and C 1s, N 1s for MXene@PDA. (B-C) Chemical structures and synthesis schematics of OHA-PBA and OHA-PBA/ CMCS. (D-E) Representative live/dead staining images and quantitative analysis of HUVECs treated with OC-MP and OCS-MPC. Scale bar = 200 μm. n = 3. All data are representative of at least three independent experiments. Data are presented as mean ± SEM. ∗P < 0.05, ∗∗P < 0.01, ∗∗∗P < 0.001, ∗∗∗P < 0.0001. Figure S2. In Vitro Osteogenic, Angiogenic, and Neurogenic Properties of OCS-MPC (A) qPCR analysis of osteogenic-related mRNA (OCN, COL-1, and OPN) expression in BMSCs cultured with OC-MP and OCS-MPC (n = 3). (B-C) Representative Transwell migration assay images and quantitative analysis of HUVECs treated with OC-MP and OCS-MPC for 24 h (scale bar = 200 μm, n = 3). (D) Quantitative analysis of tube formation assay of HUVECs treated with OC-MP and OCS-MPC (n = 3). (E) qPCR analysis of angiogenesis-related mRNA (VEGF, CD31, and HIF-1α) expression in HUVECs cultured with OC-MP and OCS-MPC (n = 3). All data are representative of at least three independent experiments. Data are presented as mean ± SEM. ∗P < 0.05, ∗∗P < 0.01, ∗∗∗P < 0.001, ∗∗∗∗P < 0.0001. Figure S3. OCS-MPC Promotes Callus Formation and Remodeling in Osteoporotic Fracture Healing (A) Representative micro-CT images of femoral fractures in mice treated with OC-MP or OCS-MPC at 1-week post-fracture. Scale bar = 1 mm. n = 6. (B) Representative Masson-stained images of femoral fractures in WT mice treated with OC-MP or OCS-MPC at 1-week post-fracture. Upper panels show global views; lower panels show close-ups of the fracture sites. Scale bar = 1 mm. (C) Immunohistochemical staining images of Col-2 protein levels in calluses from mice treated with OC-MP or OCS-MPC at 1-week post-fracture. Scale bar = 1 mm. (D) Representative micro-CT images of femoral fractures in mice treated with OC-MP or OCS-MP [file mmc1.zip › Figure s1.tif]

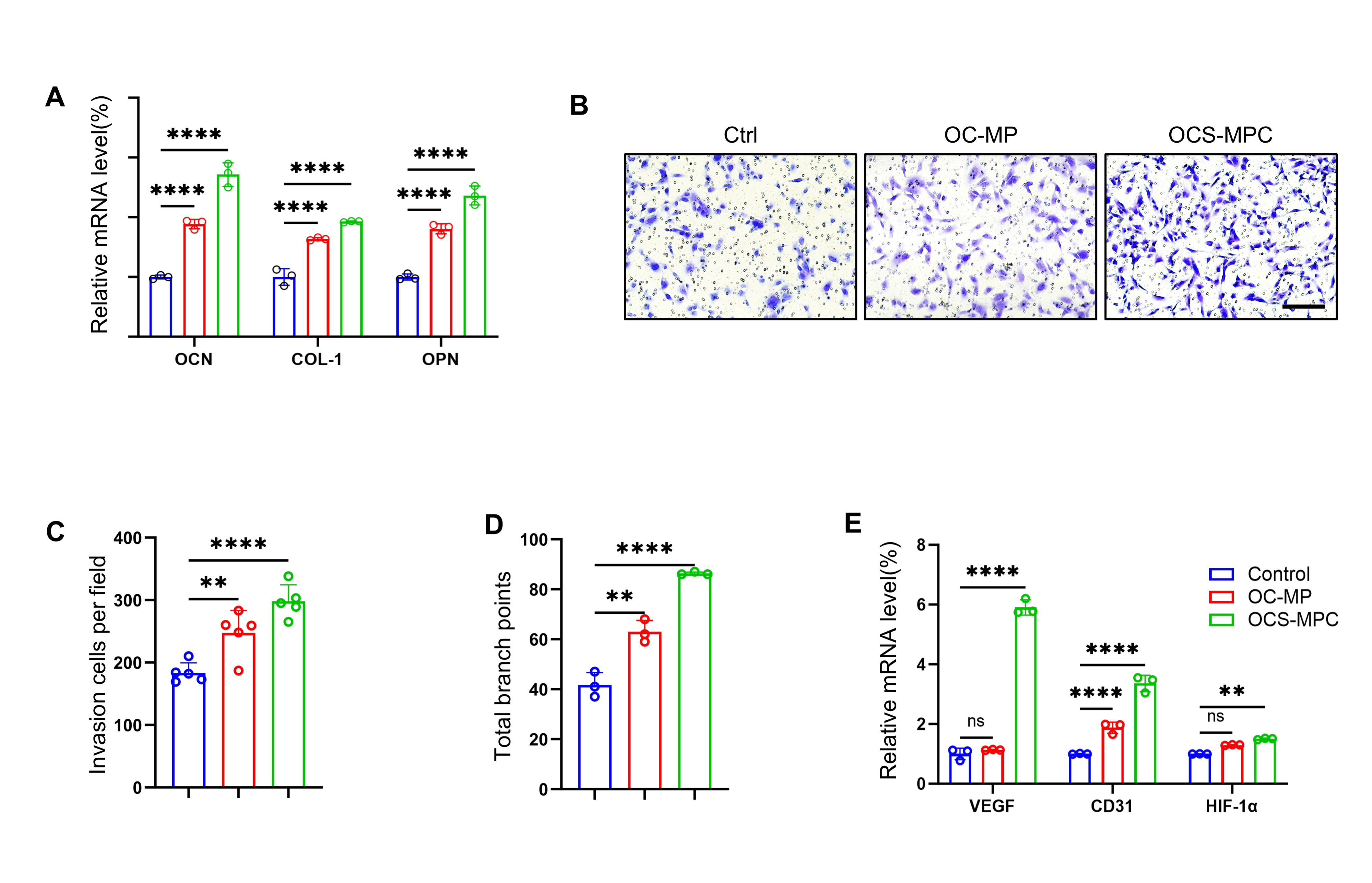

Supplement: Supplementary file 1 — Figure S1. Characterization and Properties of OCS-MPC Hydrogel (A) High-resolution XPS spectra of C 1s for MXene and C 1s, N 1s for MXene@PDA. (B-C) Chemical structures and synthesis schematics of OHA-PBA and OHA-PBA/ CMCS. (D-E) Representative live/dead staining images and quantitative analysis of HUVECs treated with OC-MP and OCS-MPC. Scale bar = 200 μm. n = 3. All data are representative of at least three independent experiments. Data are presented as mean ± SEM. ∗P < 0.05, ∗∗P < 0.01, ∗∗∗P < 0.001, ∗∗∗P < 0.0001. Figure S2. In Vitro Osteogenic, Angiogenic, and Neurogenic Properties of OCS-MPC (A) qPCR analysis of osteogenic-related mRNA (OCN, COL-1, and OPN) expression in BMSCs cultured with OC-MP and OCS-MPC (n = 3). (B-C) Representative Transwell migration assay images and quantitative analysis of HUVECs treated with OC-MP and OCS-MPC for 24 h (scale bar = 200 μm, n = 3). (D) Quantitative analysis of tube formation assay of HUVECs treated with OC-MP and OCS-MPC (n = 3). (E) qPCR analysis of angiogenesis-related mRNA (VEGF, CD31, and HIF-1α) expression in HUVECs cultured with OC-MP and OCS-MPC (n = 3). All data are representative of at least three independent experiments. Data are presented as mean ± SEM. ∗P < 0.05, ∗∗P < 0.01, ∗∗∗P < 0.001, ∗∗∗∗P < 0.0001. Figure S3. OCS-MPC Promotes Callus Formation and Remodeling in Osteoporotic Fracture Healing (A) Representative micro-CT images of femoral fractures in mice treated with OC-MP or OCS-MPC at 1-week post-fracture. Scale bar = 1 mm. n = 6. (B) Representative Masson-stained images of femoral fractures in WT mice treated with OC-MP or OCS-MPC at 1-week post-fracture. Upper panels show global views; lower panels show close-ups of the fracture sites. Scale bar = 1 mm. (C) Immunohistochemical staining images of Col-2 protein levels in calluses from mice treated with OC-MP or OCS-MPC at 1-week post-fracture. Scale bar = 1 mm. (D) Representative micro-CT images of femoral fractures in mice treated with OC-MP or OCS-MP [file mmc1.zip › Figure s2.tif]
